# Supplementary material for: Dynamics of competing SARS-CoV-2 variants during the Omicron epidemic in England
Source: Nat Commun. 2022 Jul 28;13:4375. doi: 10.1038/s41467-022-32096-4 (PMC9330949; doi:10.1038/s41467-022-32096-4)
Supplement: Supplementary file 1 — Supplementary Information [file 41467_2022_32096_MOESM1_ESM.pdf]

Supplementary figures

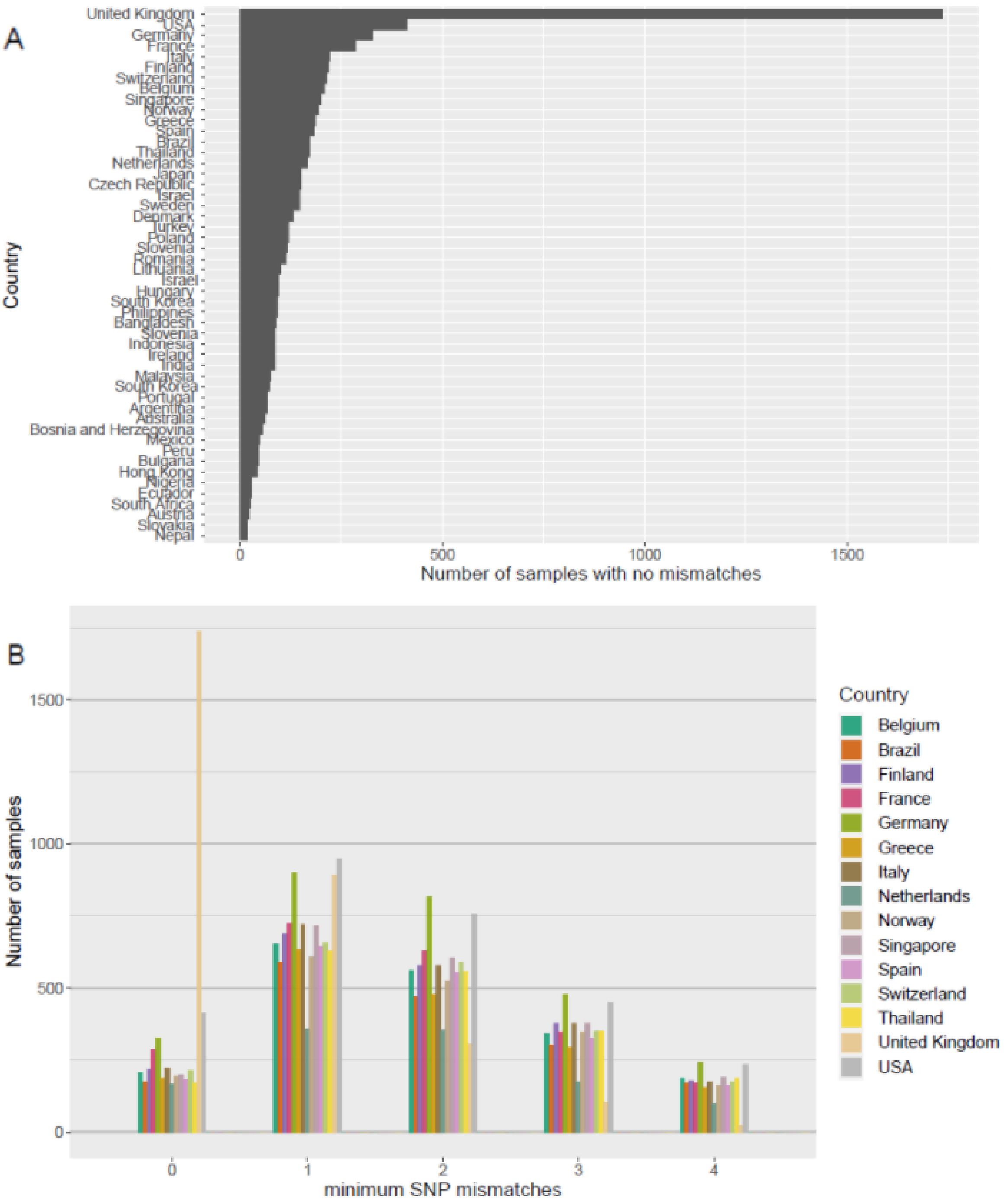

Supplementary Figure 1: Similarity of REACT-1 sequences to international sequences (A) The number of REACT-1 sequences that match (no different SNPs) a sequence obtained from GISAID by the country the sequence was sampled in. The top 50 most represented countries have been shown. (B) The number of REACT-1 sequences at a given SNP distant from sequences obtained from GISAID by the country the sequence was sampled in. The top 15 most represented countries have been shown.

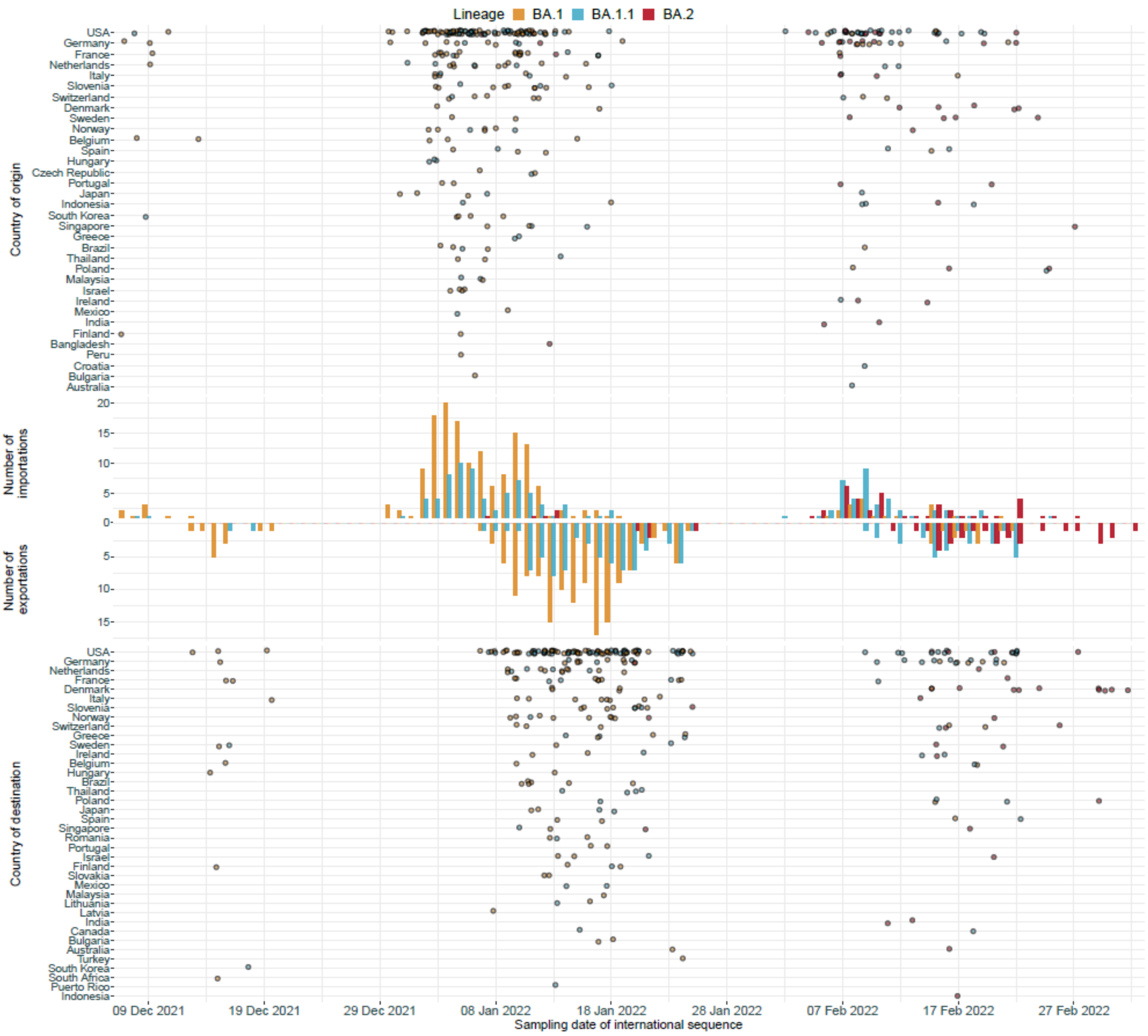

Supplementary Figure 2: Importation and exportations of Omicron. The daily number of potential importations/exportations (bars) of Omicron to/from England, as inferred with REACT-1 samples and a representative selection of global samples, for BA.1 (orange), BA.1.1 (blue) and BA.2 (red). For each importation/exportation the date of the importation/exportation and the country of the global sample (and thus the origin/destination of the Omicron strain) has been shown (dots).

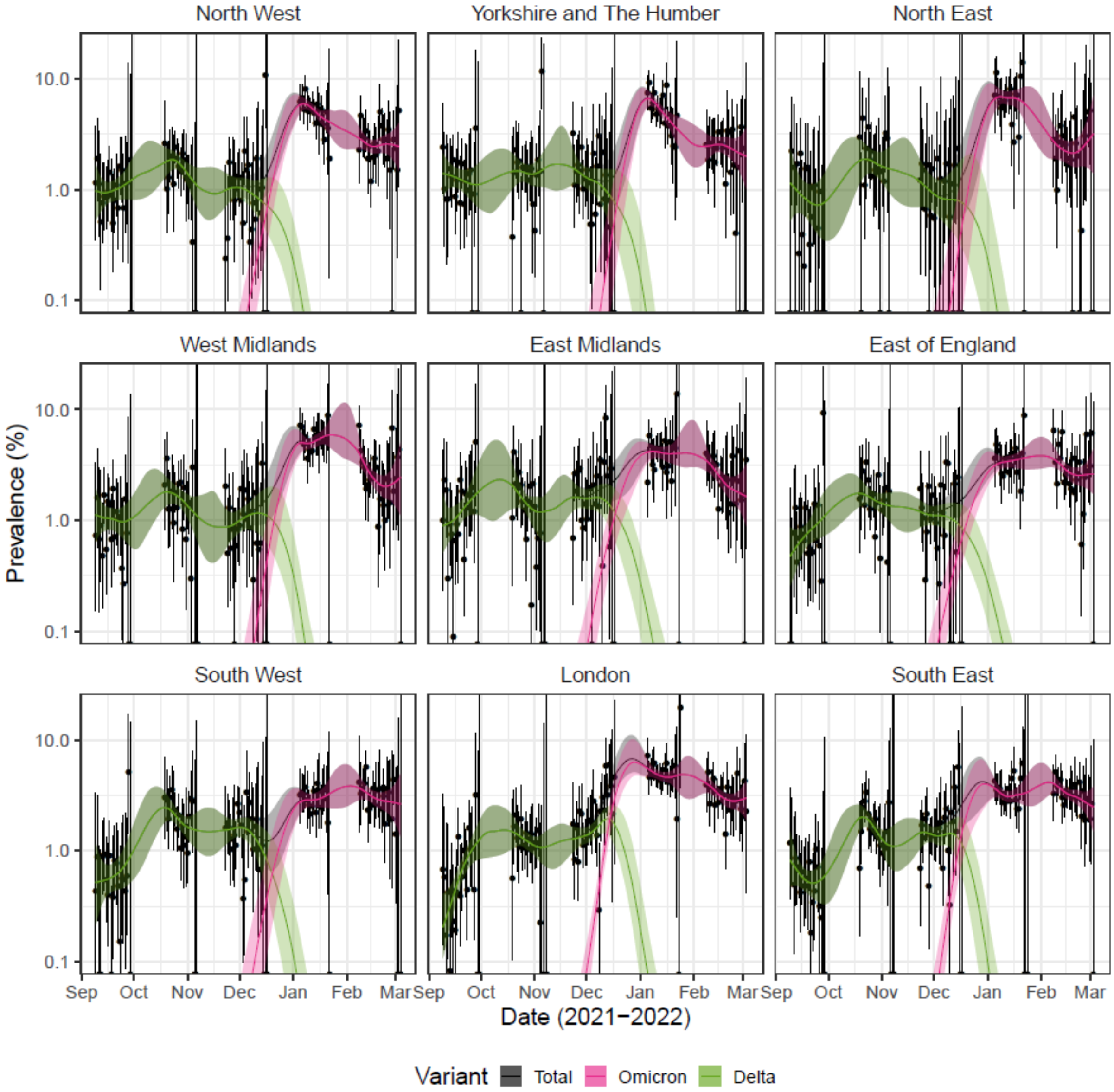

Supplementary Figure 3: Omicron vs Delta prevalence by region Modelled prevalence of SARS-CoV-2 variants Omicron (pink) and Delta (green), and total prevalence (grey) in each region of England estimated using mixed-effects Bayesian P-spline models. Estimates of prevalence are shown with a central estimate (solid line) and 95% (shaded region) credible intervals. Daily weighted estimates of mean prevalence (points) are shown with 95% credible intervals (error bars).

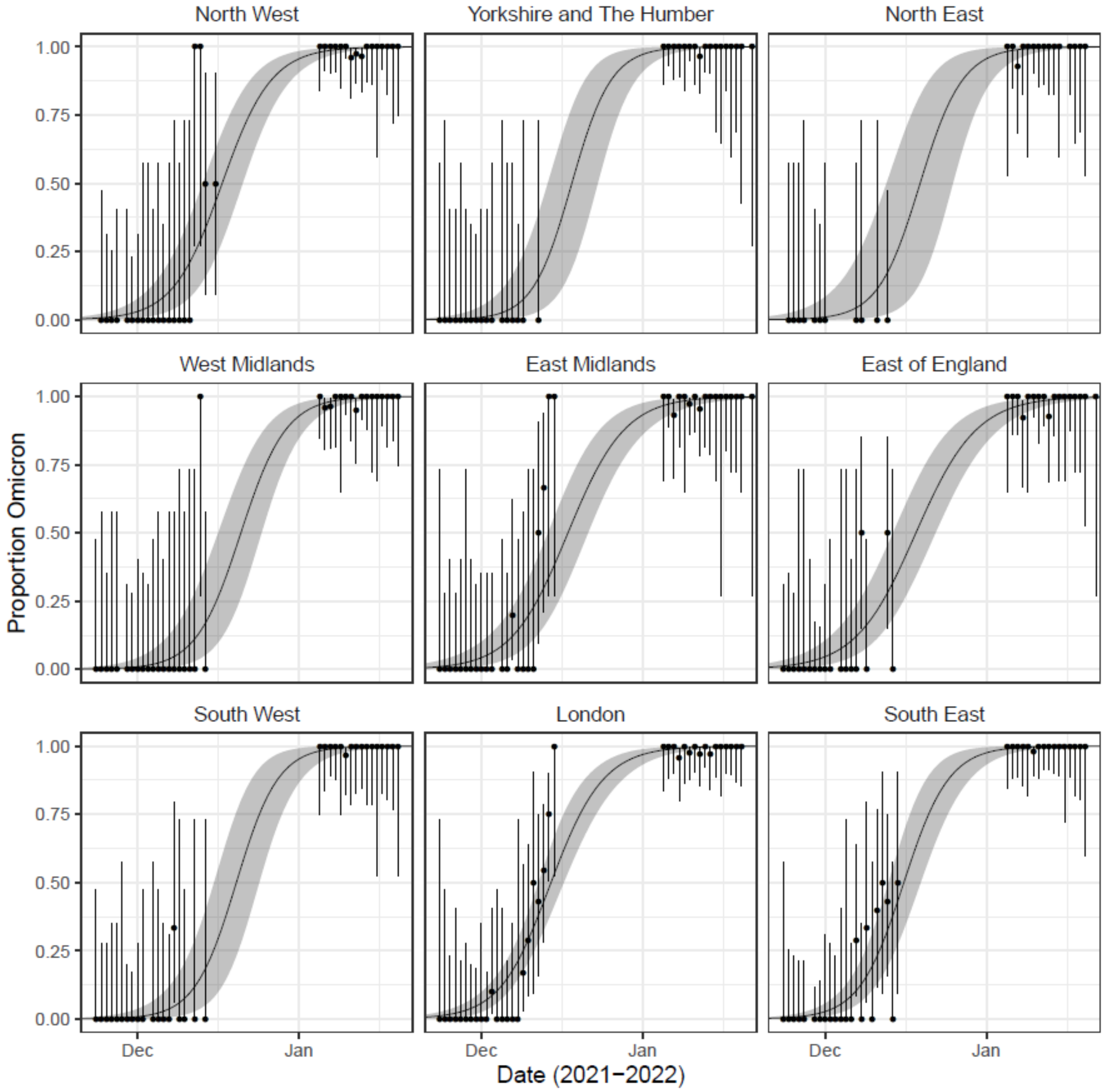

Supplementary Figure 4: Omicron vs Delta proportion by region Modelled proportions of lineages identified as Omicron in each region of England estimated using mixed-effects Bayesian P-spline models. Estimates are shown with a central estimate (solid line) and 95% credible intervals (shaded region). Daily estimates of the mean proportion of lineages Omicron (points) are shown with 95% confidence intervals (error bars).

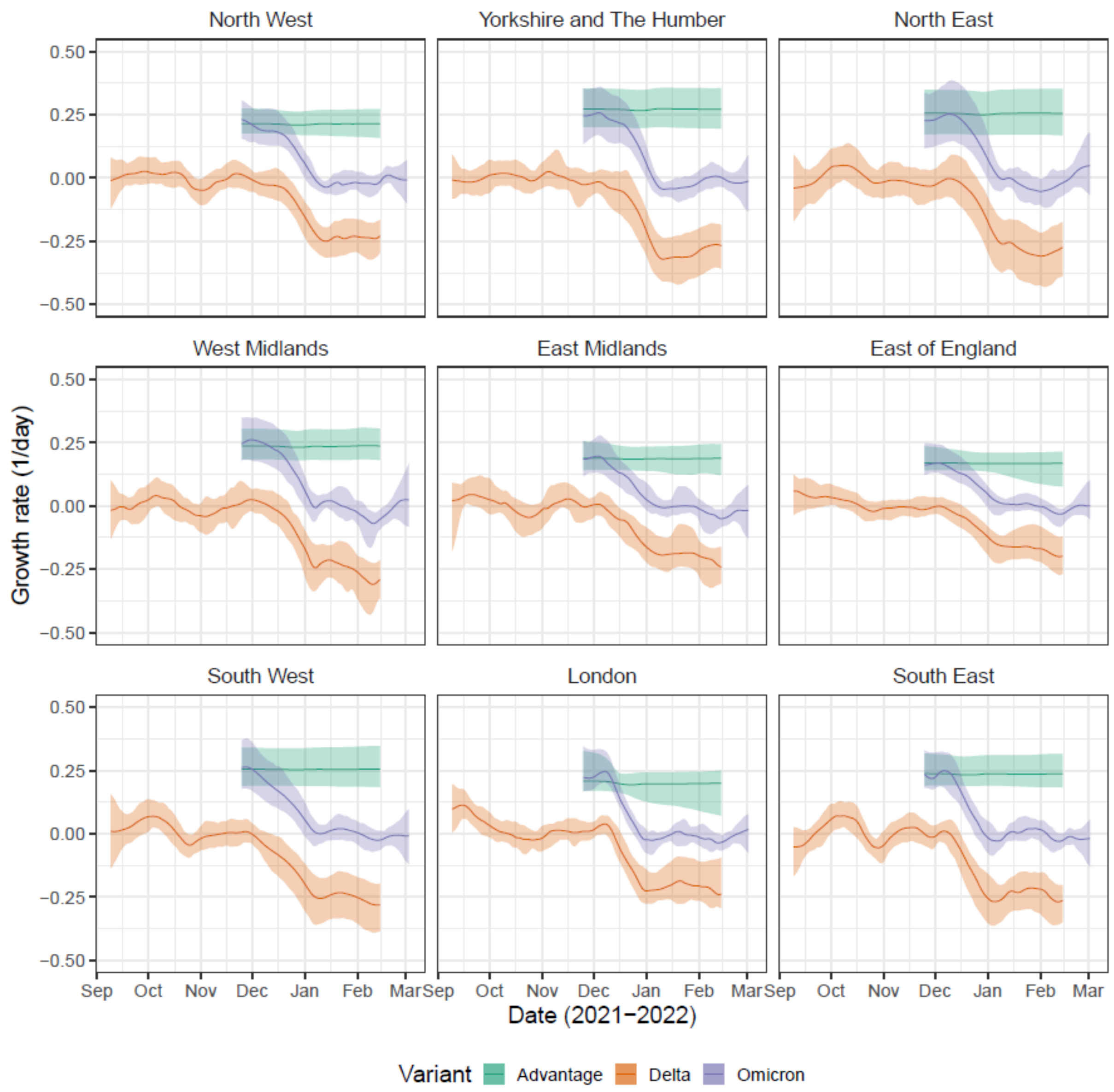

Supplementary Figure 5: Omicron vs Delta growth rate by region Daily growth rate of Omicron (purple), Delta (orange) and their additive difference (green) estimated from mixed-effects Bayesian P-spline models fitted to each region of England. Estimates are shown with a central estimate (solid line) and 95% credible intervals (shaded region). Estimates for each lineage are only displayed for the period over which the lineage was detected in REACT-1 samples.

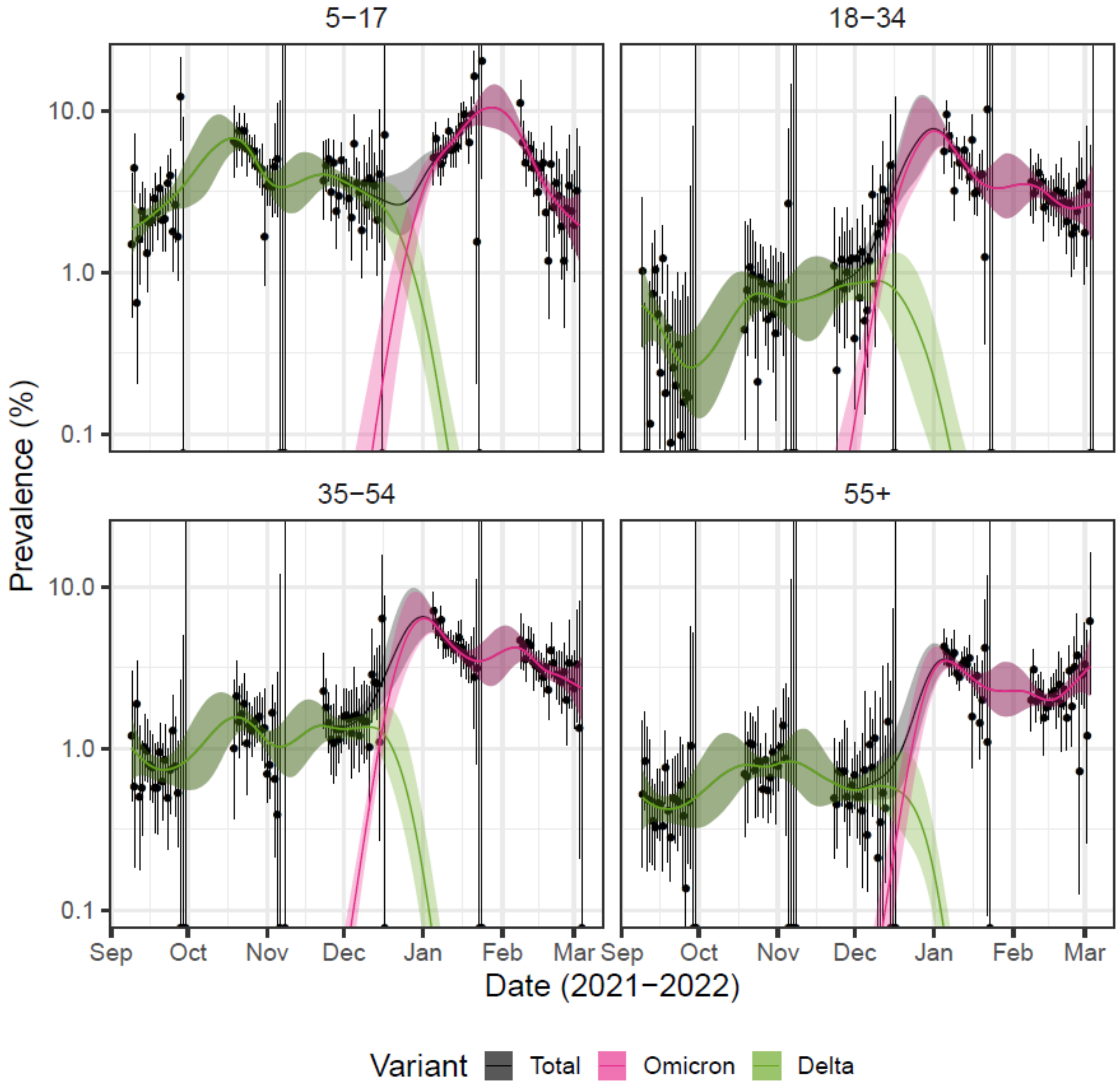

Supplementary Figure 6: Omicron vs Delta prevalence by age-group Modelled prevalence of SARS-CoV-2 variants Omicron (pink) and Delta (green), and total prevalence (grey) for each age-group in England estimated using mixed-effects Bayesian P-spline models. Estimates of prevalence are shown with a central estimate (solid line) and 95% (shaded region) credible intervals. Daily weighted estimates of mean prevalence (points) are shown with 95% credible intervals (error bars).

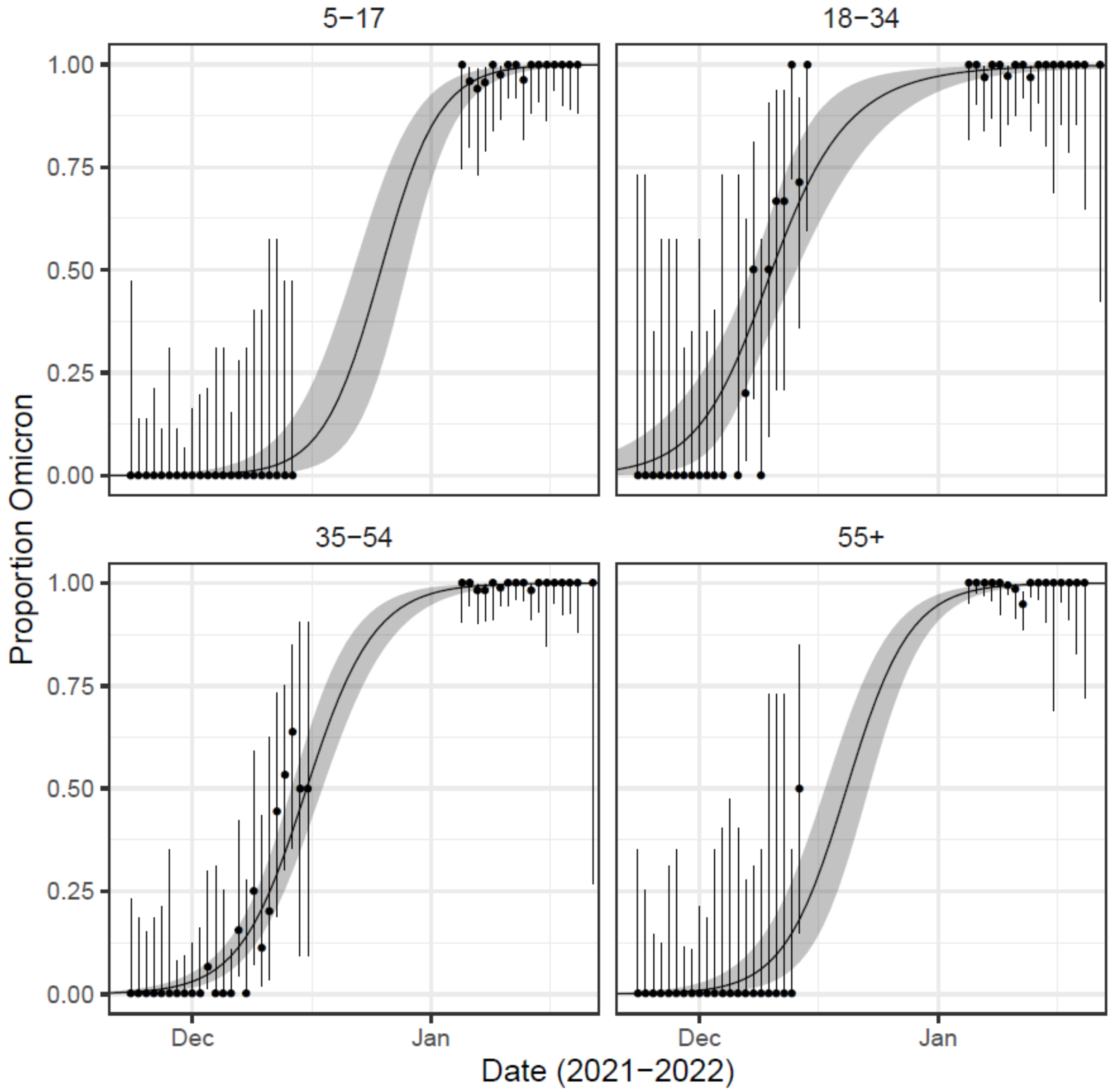

Supplementary Figure 7: Omicron vs Delta proportion by age-group Modelled proportions of lineages identified as Omicron for each age-group in England estimated using mixed-effects Bayesian P-spline models. Estimates are shown with a central estimate (solid line) and 95% credible intervals (shaded region). Daily estimates of the mean proportion of lineages Omicron (points) are shown with 95% credible intervals (error bars).

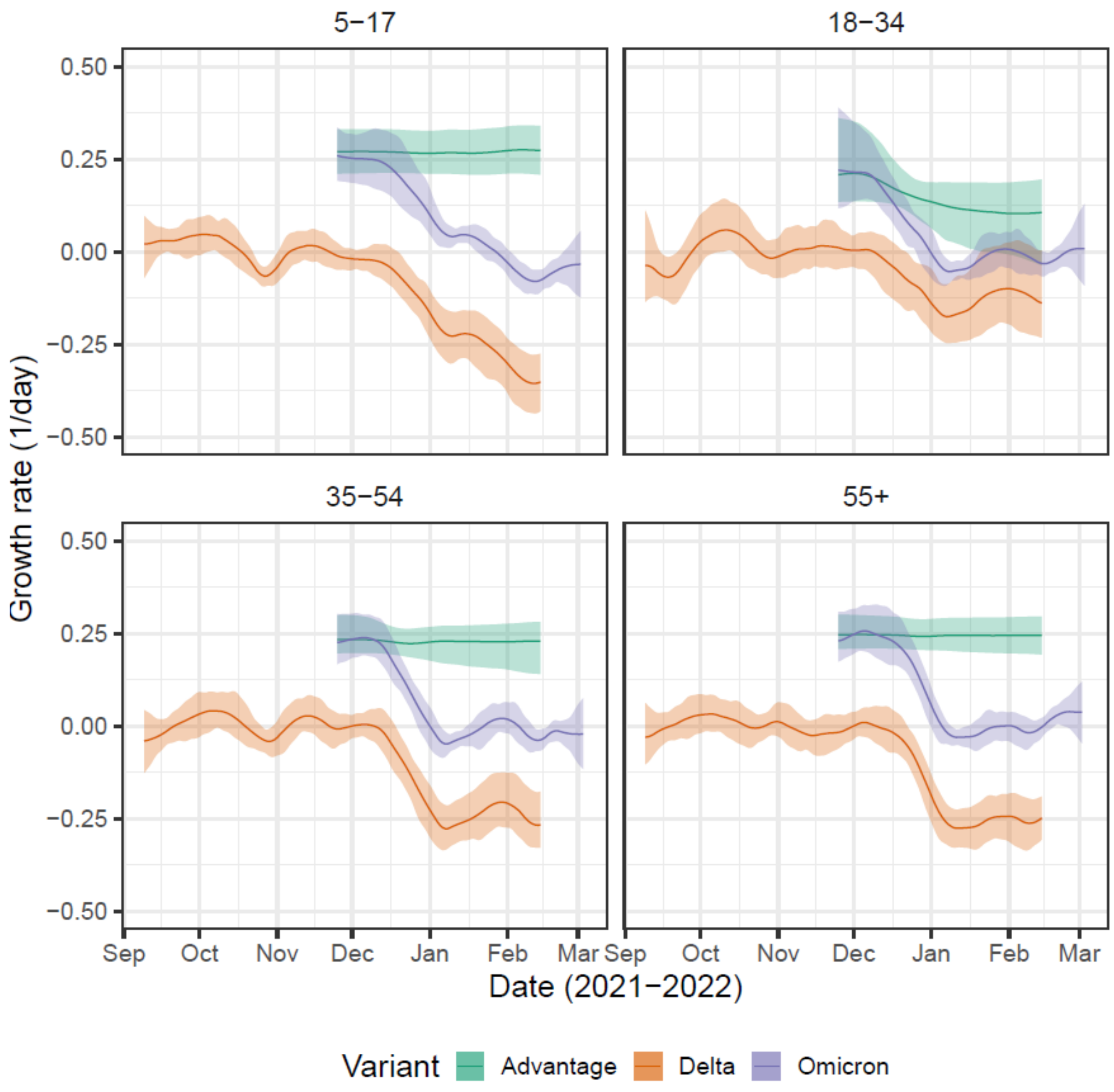

Supplementary Figure 8: Omicron vs Delta growth rate by age-group Daily growth rate of Omicron (purple), Delta (orange) and their additive difference (green) estimated from mixed-effects Bayesian P-spline models fitted to each age-group in England. Estimates are shown with a central estimate (solid line) and 95% credible intervals (shaded region). Estimates for each lineage are only displayed for the period over which the lineage was detected in REACT-1 samples.

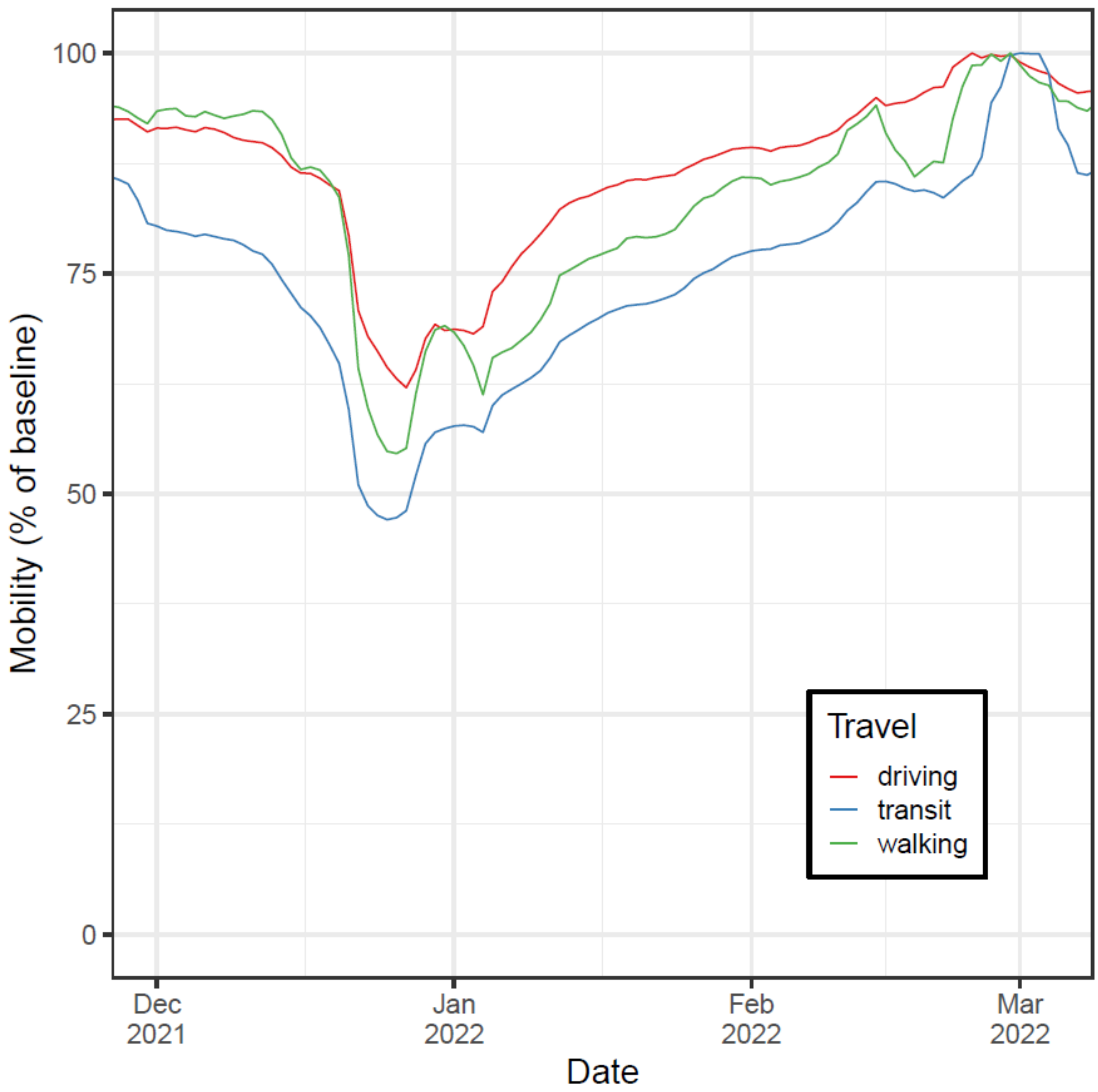

Supplementary Figure 9: Apple mobility indices Rolling 7-day average apple mobility indices in England, calculated from phone location data, for driving (red), transit (blue) and walking (green). Mobility indices have been scaled so that the maximum value reached in the period shown takes a value of 100.

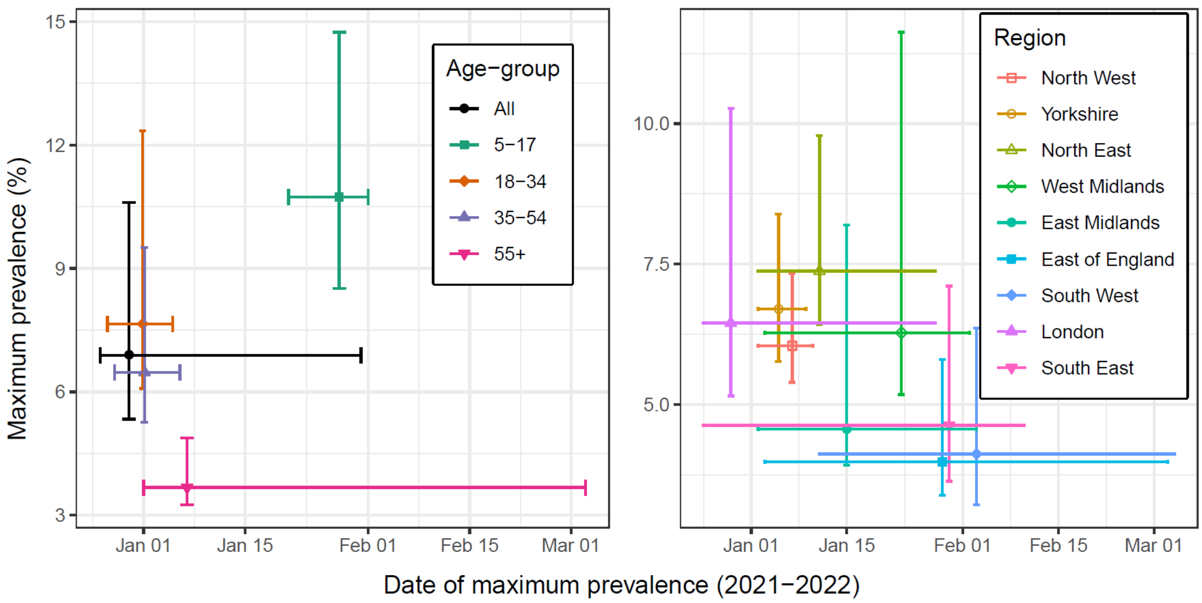

Supplementary Figure 10: Maximum prevalence and date of maximum prevalence The maximum prevalence of Omicron reached and the date at which it was reached inferred from the mixed-effects Bayesian P-spline model fitted to all data, and for the models fit to subsets of data by region and age-group. Estimates are shown for the median value (points) with 95% credible intervals (error-bars).

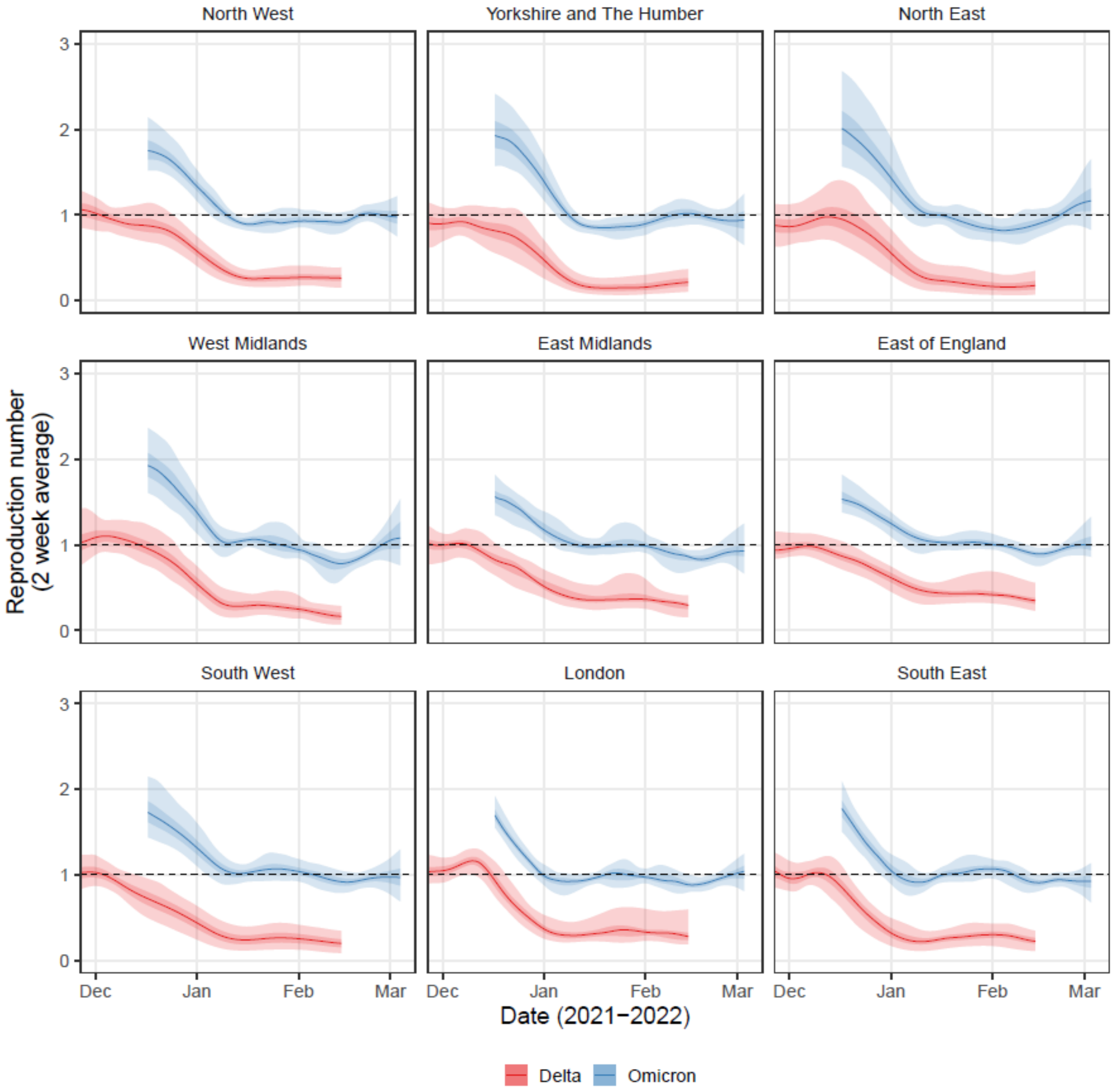

Supplementary Figure 11: Omicron vs Delta R_t by region Rolling two-week average (prior two weeks) Reproduction number for Omicron (blue) and Delta (red) in each region of England as inferred from mixed-effects Bayesian P-spline models. Estimates are shown with a central estimate (solid line) and 50% (dark shaded region) and 95% (light shaded region) credible intervals. Dashed line shows R=1 the threshold for epidemic growth. Estimates for each lineage are only displayed for the period over which the lineage was detected in REACT-1 samples.

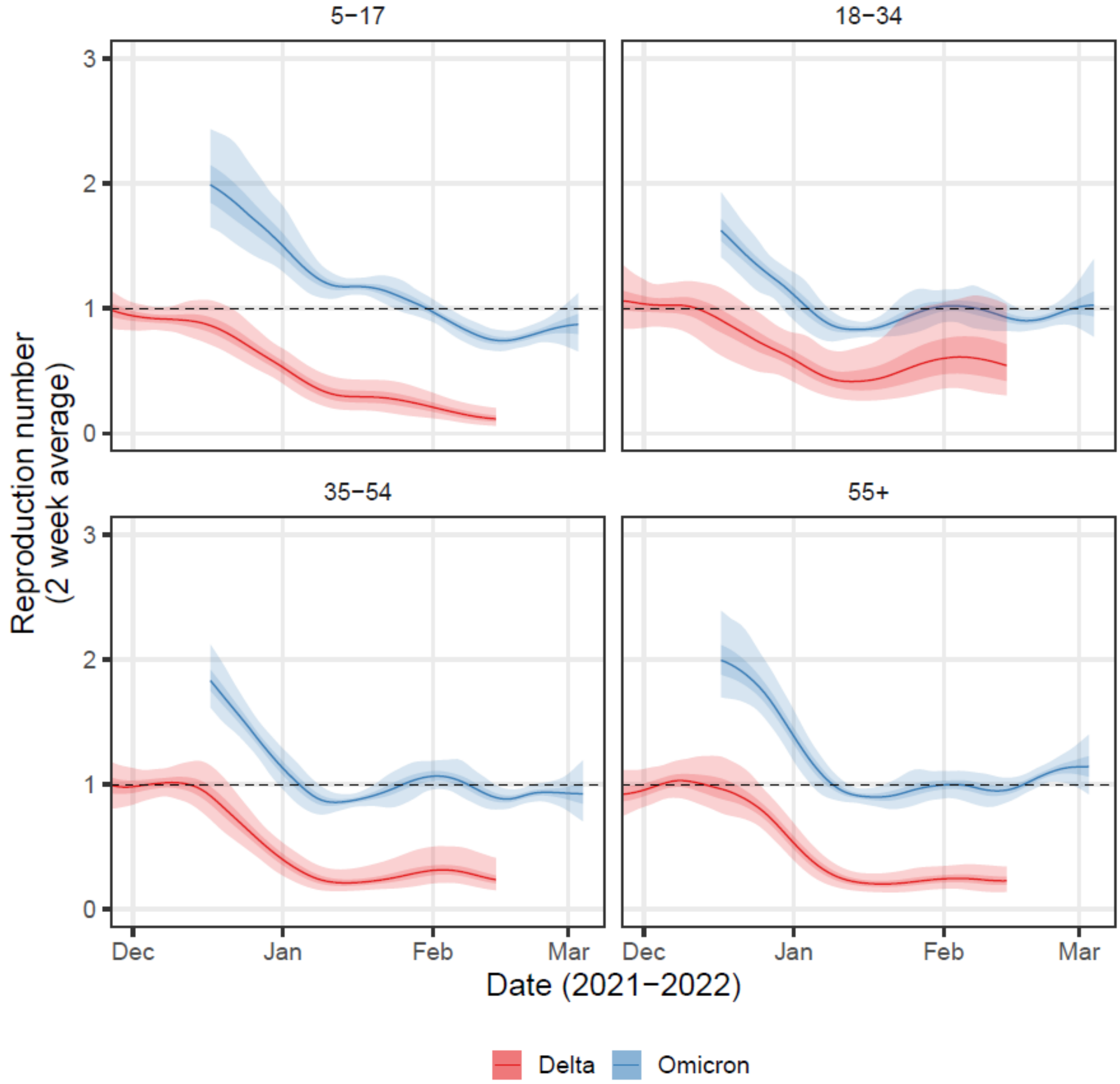

Supplementary Figure 12: Omicron vs Delta R_t by age-group Rolling two-week average (prior two weeks) Reproduction number for Omicron (blue) and Delta (red) for each age-group in England as inferred from mixed-effects Bayesian P-spline models. Estimates are shown with a central estimate (solid line) and 50% (dark shaded region) and 95% (light shaded region) credible intervals. Dashed line shows R=1 the threshold for epidemic growth. Estimates for each lineage are only displayed for the period over which the lineage was detected in REACT-1 samples.

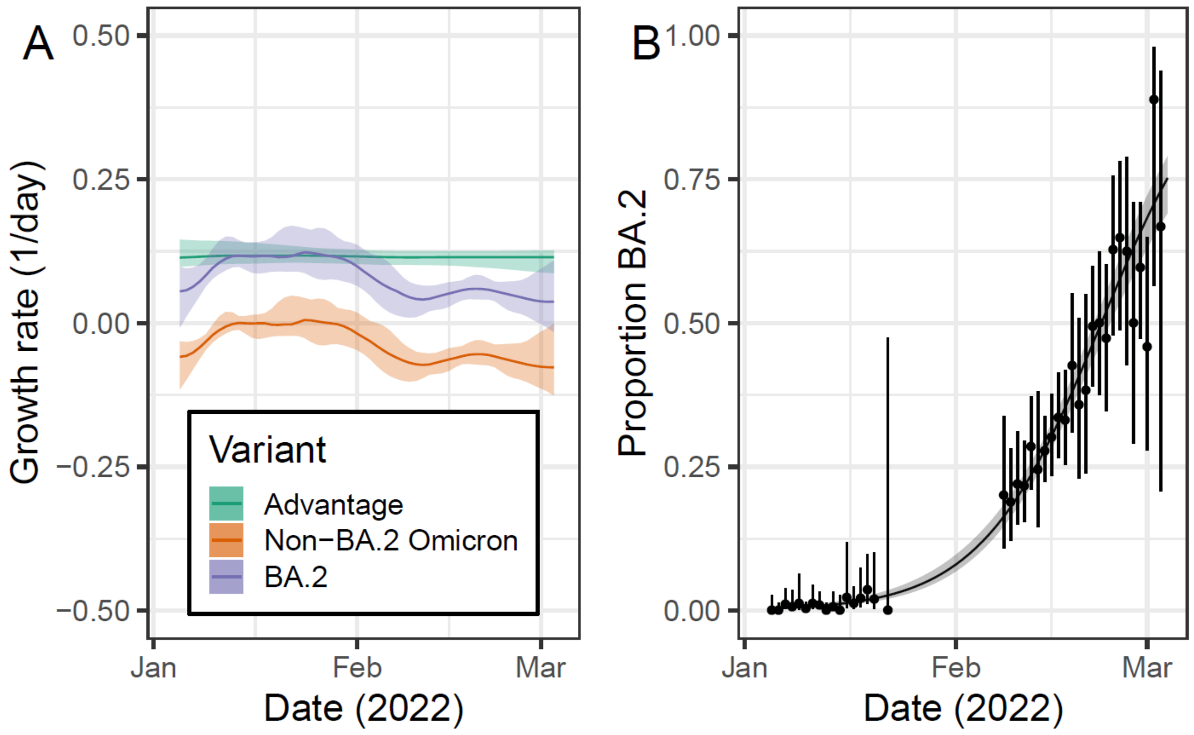

Supplementary Figure 13: BA.2 vs non-BA.2 National growth rate and proportion graph A) Daily growth rate of BA.2 (purple), non-BA.2 Omicron (orange) and their additive difference (green) estimated from the mixed-effects Bayesian P-spline model fitted to rounds 17 and 18 of the data. Estimates are shown with a central estimate (solid line) and 95% credible intervals (shaded region). B) Modelled proportion of lineages identified as BA.2 in England estimated using a mixed-effects Bayesian P-spline model. Estimates are shown with a central estimate (solid line) and 95% credible intervals (shaded region). Daily estimates of the mean proportion of lineages BA.2 (points) are shown with 95% confidence intervals (error bars).

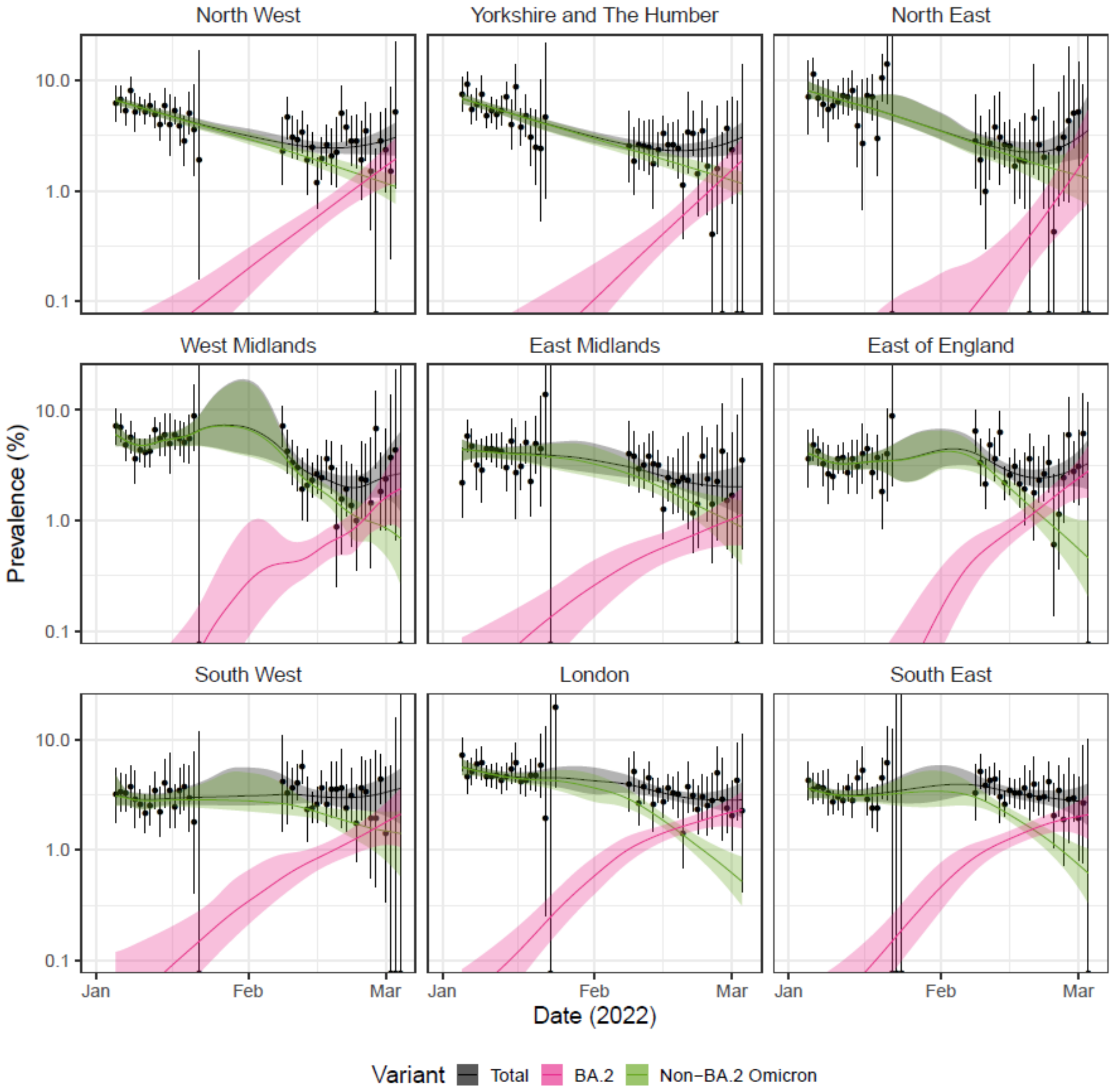

Supplementary Figure 14: BA.2 vs non-BA.2 prevalence by region Modelled prevalence of BA.2 (pink), non-BA.2 Omicron (green) and total prevalence (grey) in each region of England for rounds 17 and 18 estimated using mixed-effects Bayesian P-spline models. Estimates of prevalence are shown with a central estimate (solid line) and 95% (shaded region) credible intervals. Daily weighted estimates of mean prevalence (points) are shown with 95% credible intervals (error bars).

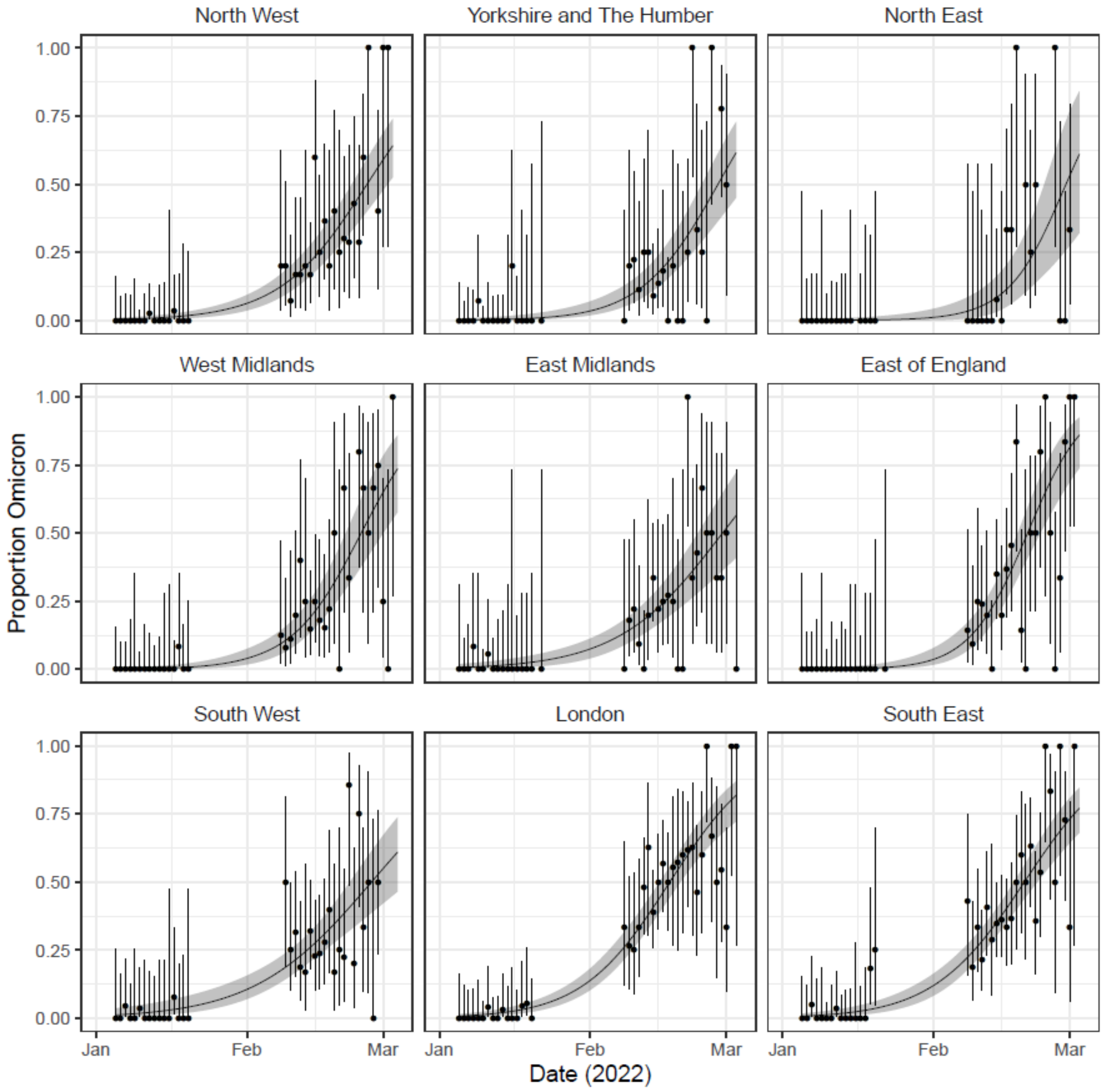

Supplementary Figure 15: BA.2 vs non-BA.2 proportion by region Modelled proportion of lineages identified as BA.2 by region of England for rounds 17 and 18 estimated using mixed-effects Bayesian P-spline models. Estimates are shown with a central estimate (solid line) and 95% credible intervals (shaded region). Daily estimates of the mean proportion of lineages BA.2 (points) are shown with 95% confidence intervals (error bars).

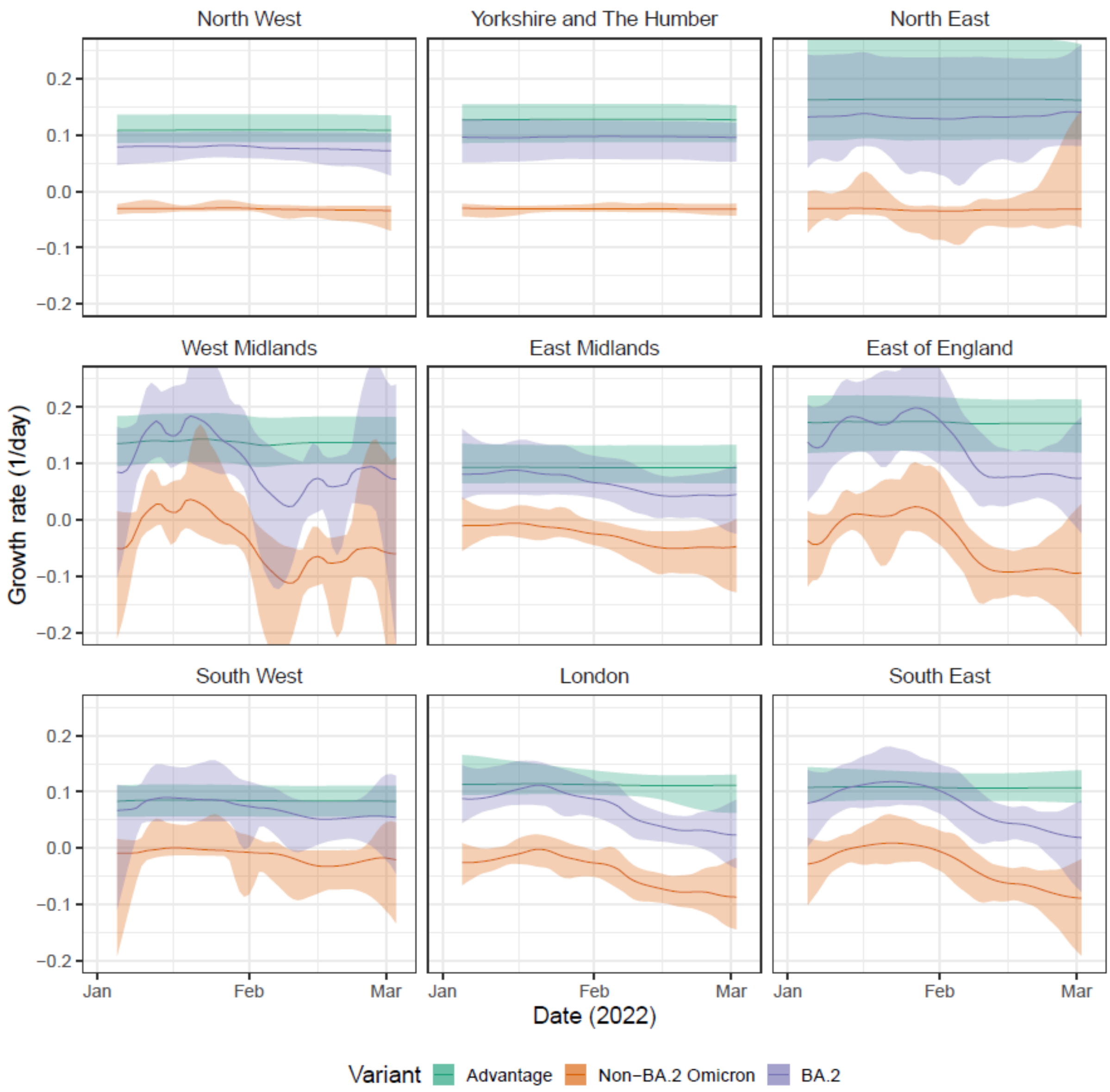

Supplementary Figure 16: BA.2 vs non-BA.2 growth rate by region Daily growth rate of BA.2 (purple), non-BA.2 Omicron (orange) and their additive difference (green) estimated from mixed-effects Bayesian P-spline models fitted for each region of England to rounds 17 and 18 of the data. Estimates are shown with a central estimate (solid line) and 95% credible intervals (shaded region).

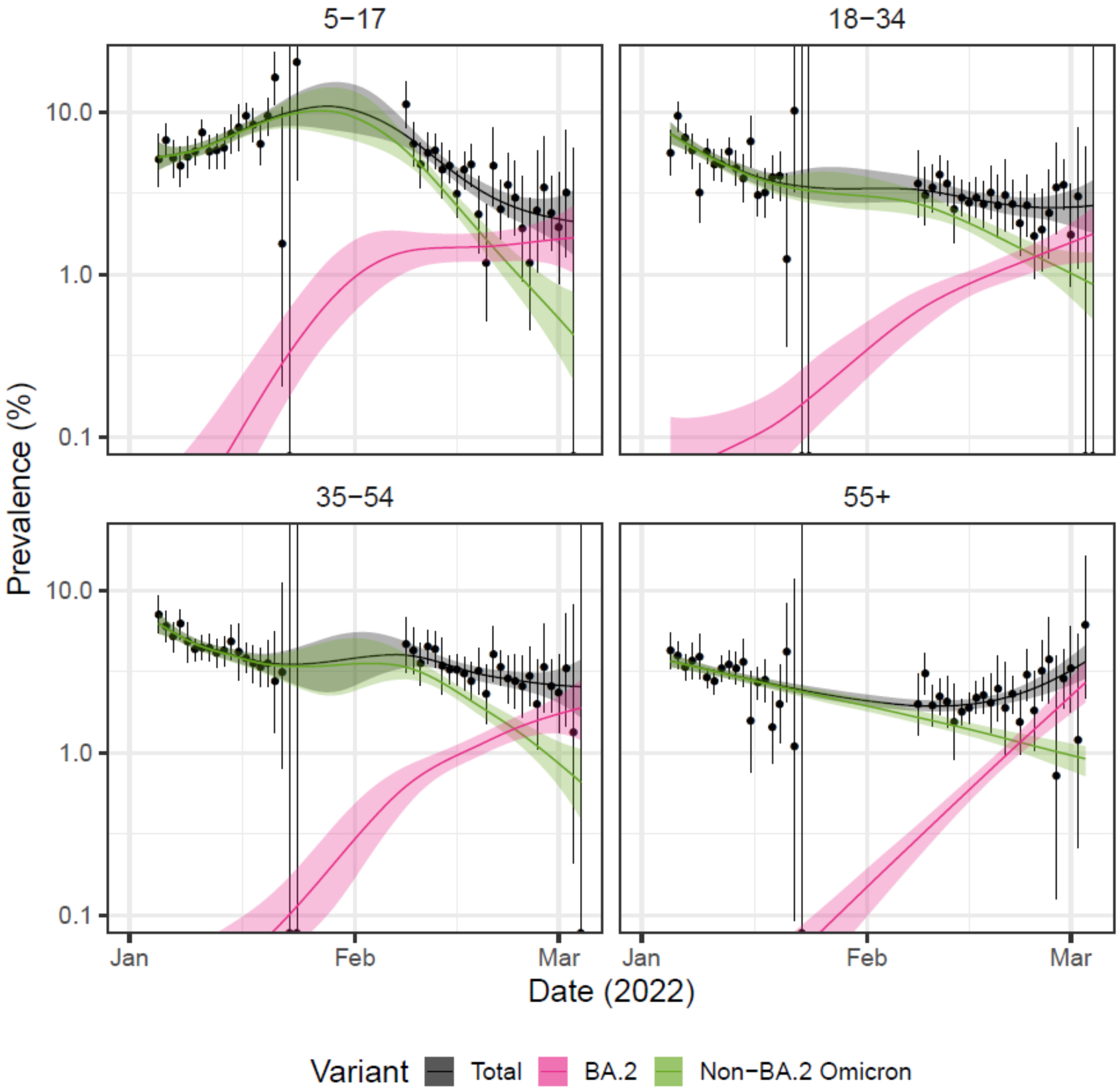

Supplementary Figure 17: BA.2 vs non-BA.2 prevalence by age-group Modelled prevalence of BA.2 (pink), non-BA.2 Omicron (green) and total prevalence (grey) for each age-group in England for rounds 17 and 18 estimated using mixed-effects Bayesian P-spline models. Estimates of prevalence are shown with a central estimate (solid line) and 95% (shaded region) credible intervals. Daily weighted estimates of mean prevalence (points) are shown with 95% credible intervals (error bars).

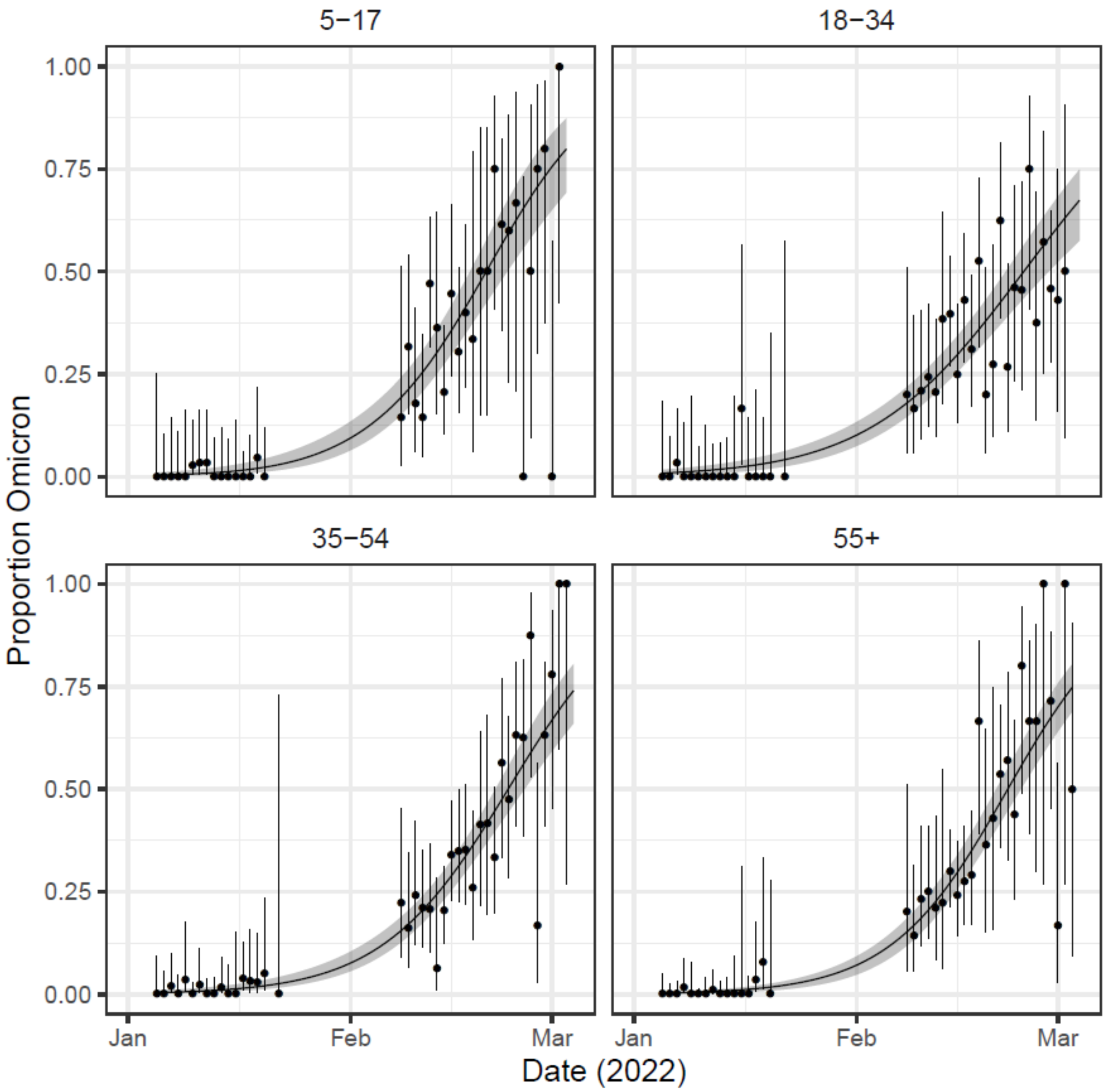

Supplementary Figure 18: BA.2 vs non-BA.2 proportion by age-group Modelled proportion of lineages identified as BA.2 by age-groups in England for rounds 17 and 18 estimated using mixed-effects Bayesian P-spline models. Estimates are shown with a central estimate (solid line) and 95% credible intervals (shaded region). Daily estimates of the mean proportion of lineages BA.2 (points) are shown with 95% confidence intervals (error bars).

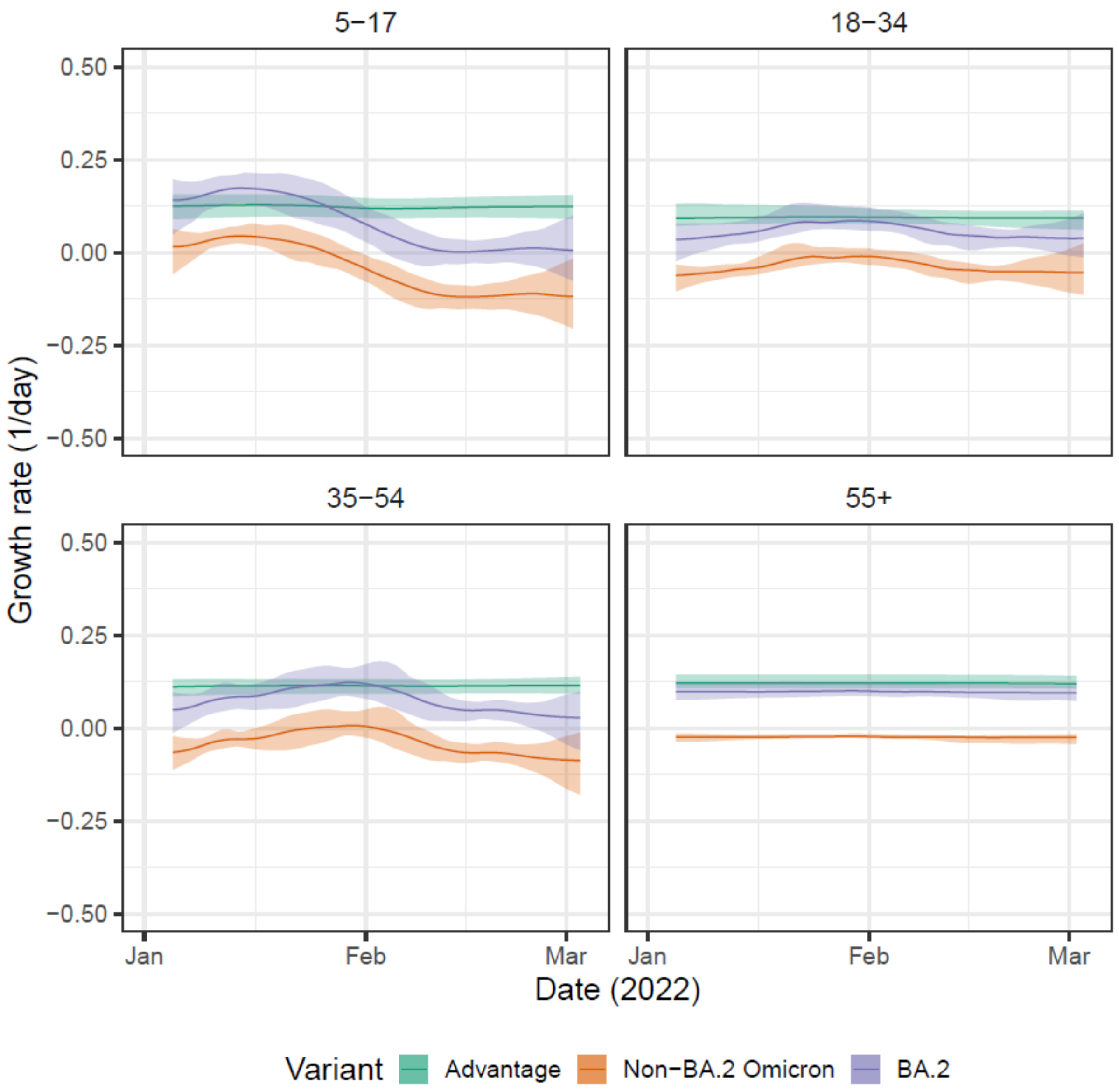

Supplementary Figure 19: BA.2 vs non-BA.2 growth rate by age-group Daily growth rate of BA.2 (purple), non-BA.2 Omicron (orange) and their additive difference (green) estimated from mixed-effects Bayesian P-spline models fitted by age-group in England to rounds 17 and 18 of the data. Estimates are shown with a central estimate (solid line) and 95% credible intervals (shaded region).

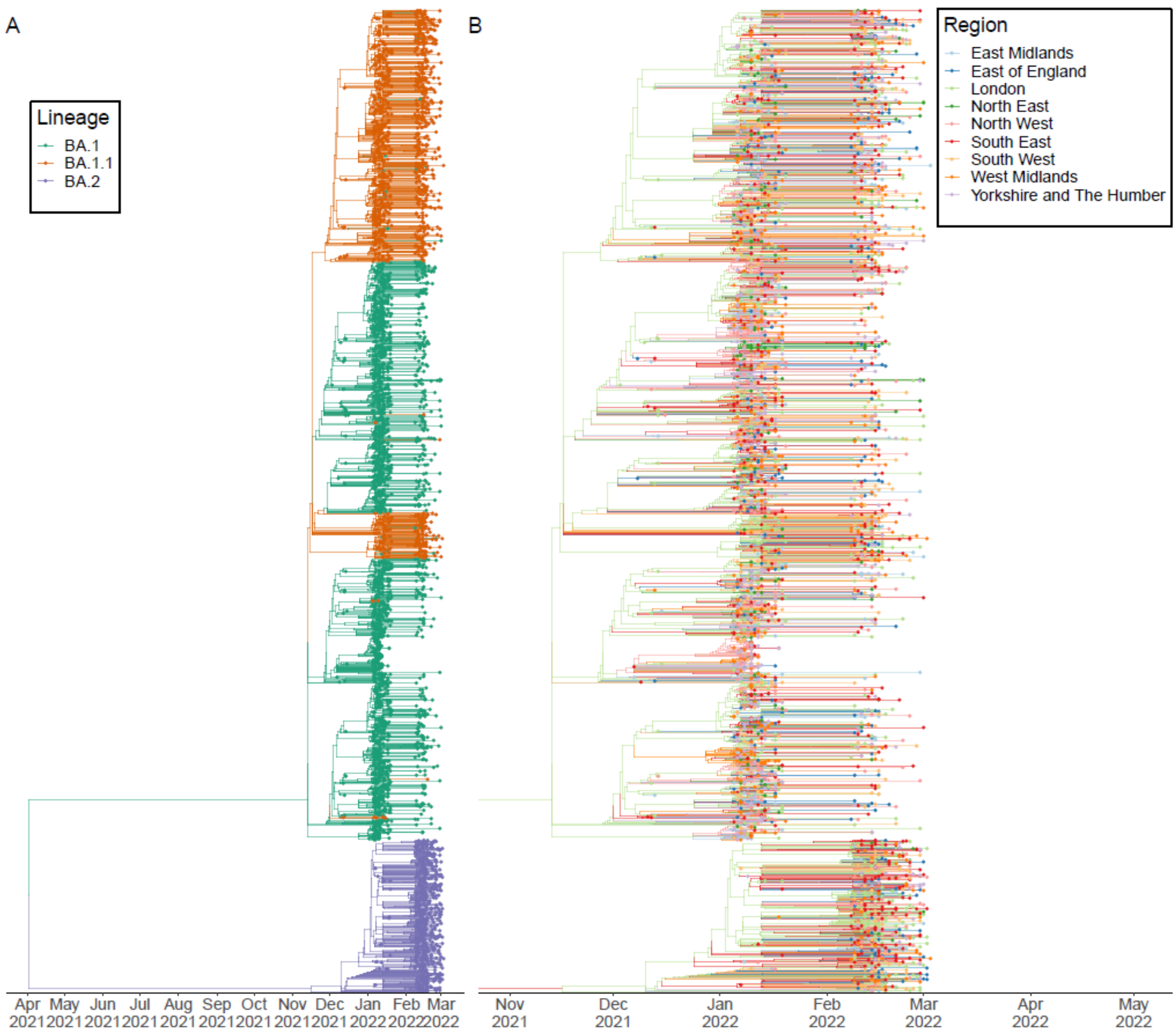

Supplementary Figure 20: Phylogeographic model of Omicron (A) Time-resolved phylogenetic tree for all Omicron lineages. Tips and nodes have been coloured by their inferred Omicron sub-lineage. (B) Time-resolved phylogenetic tree for all Omicron lineages. Tips have been coloured by the region in which they were collected. Nodes have been coloured by their inferred region (using a mugenic model). Note we have only shown the tree from November 2021 onwards.

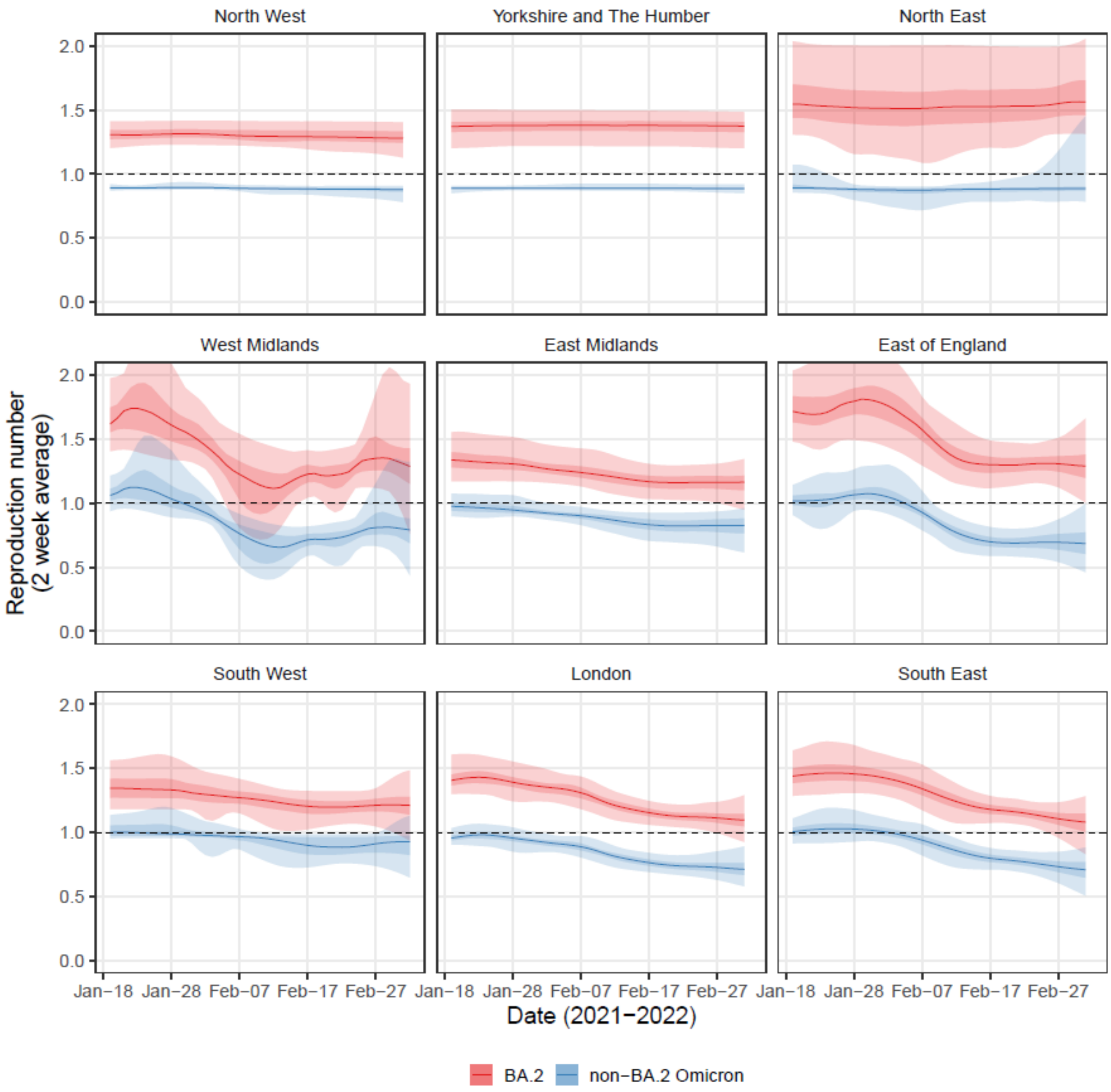

Supplementary Figure 21: BA.2 vs non-BA.2 R_t by region Rolling two-week average (prior two weeks) Reproduction number for BA.2 (red) and non-BA.2 Omicron (blue) in each region of England as inferred from mixed-effects Bayesian P-spline models fitted to rounds 17 and 18 of the data. Estimates are shown with a central estimate (solid line) and 50% (dark shaded region) and 95% (light shaded region) credible intervals. Dashed line shows R=1 the threshold for epidemic growth.

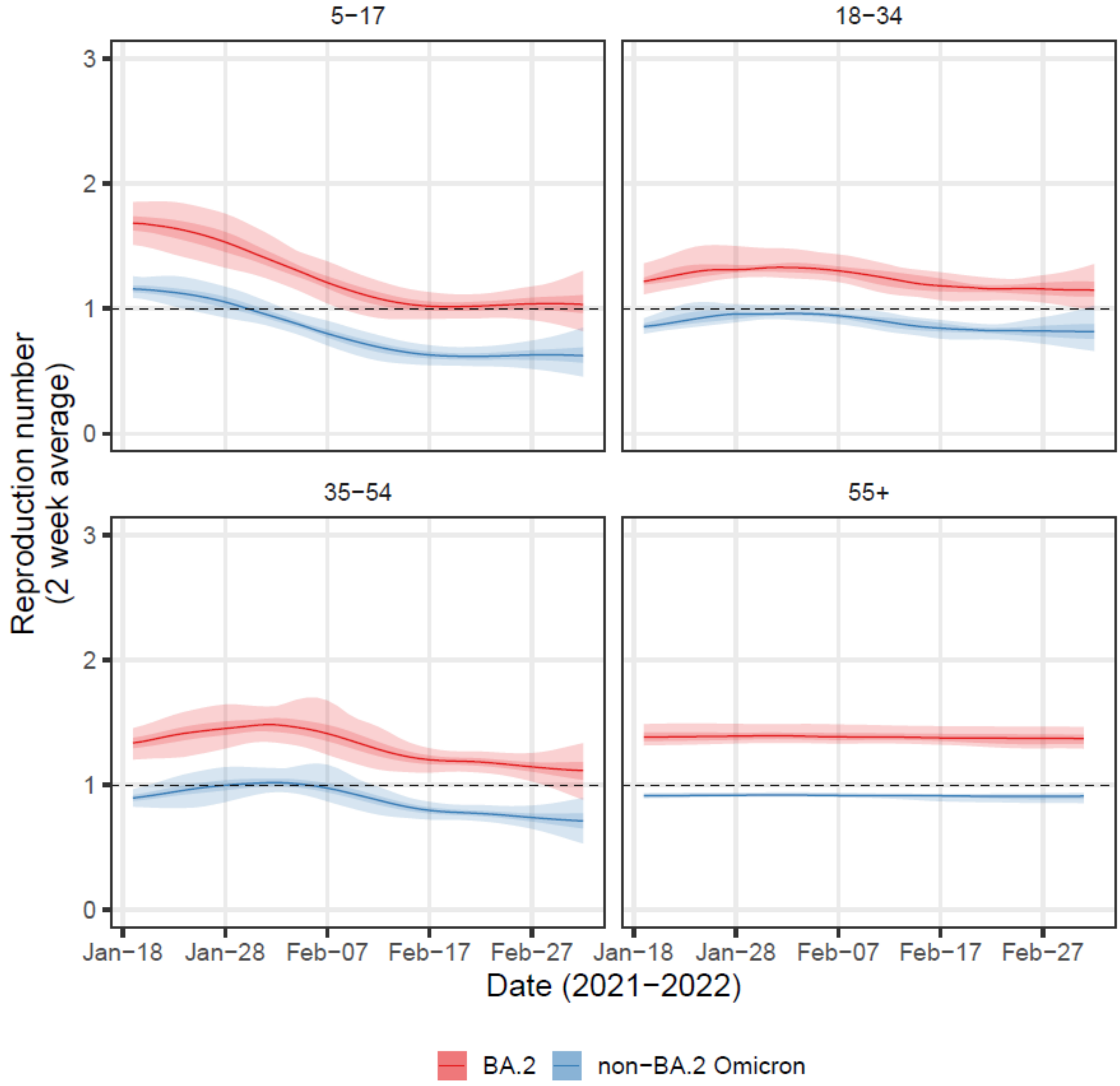

Supplementary Figure 22: BA.2 vs non-BA.2 R_t by age-group Rolling two-week average (prior two weeks) Reproduction number for BA.2 (red) and non-BA.2 Omicron (blue) for each age-group in England as inferred from mixed-effects Bayesian P-spline models fitted to rounds 17 and 18 of the data. Estimates are shown with a central estimate (solid line) and 50% (dark shaded region) and 95% (light shaded region) credible intervals. Dashed line shows R=1 the threshold for epidemic growth.

Supplementary Table 1: Estimated maximum prevalence and date of maximum prevalence for all data, and sub-groups by region and age-groups

Data	Sub-group	Maximum prevalence (95% credible intervals)	Date of maximum prevalence (95% credible intervals)
All	All	6.89% (5.34% , 10.61%)	2021-12-30 (2021-12-26 , 2022-01-31)
Region	North West	6.04% (5.39% , 7.33%)	2022-01-07 (2022-01-02 , 2022-01-10)
	North East	7.37% (6.42% , 9.79%)	2022-01-11 (2022-01-02 , 2022-01-28)
	South West	4.12% (3.21% , 6.36%)	2022-02-03 (2022-01-11 , 2022-03-01*)
	South East	4.63% (3.63% , 7.11%)	2022-01-30 (2021-12-25 , 2022-02-10)
	London	6.45% (5.15% , 10.27%)	2021-12-29 (2021-12-25 , 2022-01-28)
	East of England	3.98% (3.38% , 5.80%)	2022-01-29 (2022-01-03 , 2022-03-01*)
	East Midlands	4.56% (3.92% , 8.20%)	2022-01-15 (2022-01-02 , 2022-02-03)
	West Midlands	6.28% 5.18% 11.63%	2022-01-23 2022-01-03 2022-02-02
	Yorkshire	6.70% (5.77% , 8.39%)	2022-01-05 (2022-01-02 , 2022-01-09)
Age-group	5-17	10.74% (8.52% , 14.74%)	2022-01-28 (2022-01-21 , 2022-02-01)
	18-34	7.65% (6.08% , 12.35%)	2022-01-01 (2021-12-27 , 2022-01-05)
	35-54	6.47% (5.26% , 9.50%)	2022-01-01 (2021-12-28 , 2022-01-06)
	55+	3.67% (3.25% , 4.88%)	2022-01-07 (2022-01-01 , 2022-03-01*)

*Note that this was the last day of available data for these subsets

Supplementary Table 2: Number and proportion of each variant for rounds 16, 17 and 18

Variant	Sub-lineage	Round 16		Round 17		Round 18	
		Number	Proportion (95% confidence intervals)	Number	Proportion (95% confidence intervals)	Number	Proportion (95% confidence intervals)
Omicron	All	56	0.073 (0.056 , 0.093)	2371	0.992 (0.988 , 0.995)	1755	0.999 (0.997 , 1.000)
			Proportion of Omicron		Proportion of Omicron		Proportion of Omicron
	BA.1	47	0.839 (0.722 , 0.913)	1835	0.774 (0.757 , 0.790)	506	0.288 (0.268 , 0.310)
	BA.1.1	9	0.161 (0.087 , 0.278)	517	0.218 (0.202 , 0.235)	638	0.364 (0.341 , 0.386)
	BA.2			19	0.008 (0.005 , 0.012)	611	0.348 (0.326 , 0.371)
Delta	All	714	0.927 (0.907 , 0.944)	18	0.008 (0.005 , 0.012)	1	0.001 (0.000 , 0.003)
			Proportion of Delta		Proportion of Delta		Proportion of Delta
	AY.110	2	0.003 (0.001 , 0.010)				
	AY.111	5	0.007 (0.003 , 0.016)				
	AY.112	13	0.018 (0.011 , 0.031)				
	AY.116	1	0.001 (0.000 , 0.008)				
	AY.120	8	0.011 (0.006 , 0.022)				
	AY.121	2	0.003 (0.001 , 0.010)				
	AY.122	9	0.013 (0.007 , 0.024)				
	AY.125	2	0.003 (0.001 , 0.010)				
	AY.126	2	0.003 (0.001 , 0.010)				
	AY.127	3	0.004 (0.001 , 0.012)				
	AY.25.1	6	0.008 (0.004 , 0.018)				
	AY.33	1	0.001 (0.000 , 0.008)				
	AY.34	4	0.006 (0.002 , 0.014)				
	AY.34.1	2	0.003 (0.001 , 0.010)	1	0.056 (0.010 , 0.258)		
	AY.36	4	0.006 (0.002 , 0.014)	1	0.056 (0.010 , 0.258)		
	AY.39	6	0.008 (0.004 , 0.018)			1	1.000 (0.207 , 1.000)
	AY.4	340	0.476 (0.440 , 0.513)	7	0.389 (0.203 , 0.614)		
	AY.4.11	1	0.001 (0.000 , 0.008)				
	AY.4.15	1	0.001 (0.000 , 0.008)				
	AY.4.2	78	0.109 (0.088 , 0.134)				
	AY.4.2.1	23	0.032 (0.022 , 0.048)	1	0.056 (0.010 , 0.258)		
	AY.4.2.2	27	0.038 (0.026 , 0.054)	2	0.111 (0.031 , 0.328)		
	AY.4.2.3	3	0.004 (0.001 , 0.012)				
	AY.4.2.4	1	0.001 (0.000 , 0.008)				
	AY.4.8	7	0.010 (0.005 , 0.020)				
	AY.4.9	1	0.001 (0.000 , 0.008)				
	AY.43	39	0.055 (0.040 , 0.074)				
	AY.44	3	0.004 (0.001 , 0.012)				
	AY.46	7	0.010 (0.005 , 0.020)				
	AY.46.5	3	0.004 (0.001 , 0.012)				
	AY.46.6	2	0.003 (0.001 , 0.010)				
	AY.5	25	0.035 (0.024 , 0.051)	1	0.056 (0.010 , 0.258)		
	AY.5.6	1	0.001 (0.000 , 0.008)				
	AY.6	5	0.007 (0.003 , 0.016)				
	AY.75	2	0.003 (0.001 , 0.010)	2	0.111 (0.031 , 0.328)		
	AY.9	2	0.003 (0.001 , 0.010)				
	AY.9.2	4	0.006 (0.002 , 0.014)				
	AY.90	1	0.001 (0.000 , 0.008)				
	AY.93	1	0.001 (0.000 , 0.008)				
	AY.98	31	0.043 (0.031 , 0.061)				
	AY.98.1	3	0.004 (0.001 , 0.012)	1	0.056 (0.010 , 0.258)		
	B.1.617.2	33	0.046 (0.033 , 0.064)	2	0.111 (0.031 , 0.328)		
Wildtype	All			1	0.000 (0.000 , 0.002)		
					Proportion of wildtype		
	B.1.1.174			1	1.000 (0.207 , 1.000)		
Total		770		2390		1756	

Supplementary Table 3: Number and proportion reporting symptoms by lineage for round 16 (Omicron and Delta) and rounds 17-18 (BA.1, BA.1.1 and BA.2). P-values are calculated using a two-sided t-test. No adjustments were made for multiple comparisons.

Rounds	Lineage	Symptoms	n	N	Proportion (95% confidence intervals)	P-value
16	Delta	Any symptoms	461	641	0.719 (0.683 , 0.753)	ref
	Omicron		31	43	0.721 (0.573 , 0.833)	0.98
	Delta	Most predictive COVID-19 symptoms	312	641	0.487 (0.448 , 0.525)	ref
	Omicron		22	43	0.512 (0.368 , 0.654)	0.75
17-18	BA.1	Any symptoms	1505	2081	0.723 (0.704 , 0.742)	ref
	BA.1.1		749	1031	0.726 (0.698 , 0.753)	0.85
	BA.2		429	550	0.780 (0.744 , 0.813)	0.01
	BA.1	Most predictive COVID-19 symptoms	945	2081	0.454 (0.433 , 0.476)	ref
	BA.1.1		514	1031	0.499 (0.468 , 0.529)	0.02
	BA.2		304	550	0.553 (0.511 , 0.594)	0.00004

Supplementary Table 4: Multivariable logistic models investigating the significant differences in symptoms between BA.1 vs BA.2 and BA.1.1 when including round and N-gene Ct value as additional covariates in the model. P-values for each parameter's mean being different to the reference parameter are calculated using a two-sided t-test. No adjustments were made for multiple comparisons.

	Exhibiting most predictive COVID-19 symptoms (BA.2 vs BA.1)			Exhibiting any symptoms (BA.2 vs BA.1)			Exhibiting most predictive COVID-19 symptoms (BA.1.1 vs BA.1)		
Model	Parameters	Odds ratio (95% confidence intervals)	p-value	Parameters	Odds ratio (95% confidence intervals)	p-value	Parameters	Odds ratio	p-value
Model 1									
Antibody Positivity ~ Lineage	BA.2	1.49 (1.23 , 1.79)	0.00004	BA.2	1.36 (1.09 , 1.70)	0.01	BA.1.1	1.20 (1.03 , 1.39)	0.02
Model 2									
Antibody Positivity ~ Lineage	BA.2	1.33 (1.05 , 1.70)	0.02	BA.2	1.24 (0.93 , 1.64)	0.14	BA.1.1	1.15 (0.99 , 1.35)	0.08
+ Round	Round 18	1.15 (0.94 , 1.41)	0.17	Round 18	1.13 (0.90 , 1.42)	0.30	Round 18	1.11 (0.94 , 1.30)	0.21
Model 3									
Antibody Positivity ~ Lineage	BA.2	1.44 (1.19 , 1.74)	0.0002	BA.2	1.29 (1.03 , 1.62)	0.03	BA.1.1	1.17 (1.01 , 1.36)	0.04
+ N-gene Ct value	N-gene Ct value	0.82 (0.75 , 0.90)	0.00001	N-gene Ct value	0.74 (0.67 , 0.82)	<0.00001	N-gene Ct value	0.78 (0.71 , 0.84)	<0.00001
Model 4									
Antibody Positivity ~ Lineage	BA.2	1.27 (0.99 , 1.62)	0.06	BA.2	1.14 (0.86 , 1.52)	0.36	BA.1.1	1.12 (0.95 , 1.31)	0.18
+ Round	Round	1.19 (0.97 , 1.46)	0.10	Round	1.18 (0.93 , 1.49)	0.17	Round	1.15 (0.98 , 1.35)	0.09
+ N-gene Ct value	N-gene Ct value	0.82 (0.75 , 0.89)	0.00001	N-gene Ct value	0.74 (0.66 , 0.82)	<0.00001	N-gene Ct value	0.77 (0.71 , 0.84)	<0.00001

Odds ratio for BA.2/BA.1.1 is relative to BA.1

Odds ratio for N-gene Ct is relative to a change in Ct of +5

Odds ratio for round 18 is relative to round 17

Supplementary Table 5: Average inter-region migration rates, inferred from a mugenic model run on the time-resolved phylogenetic tree for Omicron presented for all samples by round and by Omicron sub-lineage.

	East Midlands	East of England	London	North East	North West	South East	South West	West Midlands	Yorkshire
	Total								
East Midlands	0	0.5657	2.0194	0.2859	1.1805	1.2221	0.5909	1.2403	0.934
East of England	0.5657	0	1.8615	0.438	1.1094	1.1487	0.6259	0.968	0.8763
London	2.0194	1.8615	0	1.1369	3.5052	3.8721	1.8407	2.4355	2.1362
North East	0.2859	0.438	1.1369	0	1.1419	0.7697	0.3465	0.5225	0.671
North West	1.1805	1.1094	3.5052	1.1419	0	2.1963	1.4265	1.8809	2.0835
South East	1.2221	1.1487	3.8721	0.7697	2.1963	0	1.4004	1.609	1.9281
South West	0.5909	0.6259	1.8407	0.3465	1.4265	1.4004	0	0.9699	0.5516
West Midlands	1.2403	0.968	2.4355	0.5225	1.8809	1.609	0.9699	0	1.3336
Yorkshire	0.934	0.8763	2.1362	0.671	2.0835	1.9281	0.5516	1.3336	0
	Round 16								
East Midlands	0	0.8439	2.9644	0.8158	1.128	1.1881	0.8158	1.2893	0.8158
East of England	0.8439	0	2.4442	0.8171	0.8207	1.2971	0.8171	0.8173	0.8171
London	2.9644	2.4442	0	0.7791	2.2679	4.2336	0.7791	0.8909	0.7791
North East	0.8158	0.8171	0.7791	0	0.8169	0.812	0.8193	0.8192	0.8193
North West	1.128	0.8207	2.2679	0.8169	0	0.8198	0.8169	0.8173	0.8169
South East	1.1881	1.2971	4.2336	0.812	0.8198	0	0.812	0.8135	0.812
South West	0.8158	0.8171	0.7791	0.8193	0.8169	0.812	0	0.8192	0.8193
West Midlands	1.2893	0.8173	0.8909	0.8192	0.8173	0.8135	0.8192	0	0.8192
Yorkshire	0.8158	0.8171	0.7791	0.8193	0.8169	0.812	0.8193	0.8192	0
	Round 17								
East Midlands	0	0.3657	1.716	0.2757	1.4086	1.2336	0.7676	0.9277	1.0356
East of England	0.3657	0	1.283	0.3547	1.123	1.1485	0.4644	0.99	1.6072
London	1.716	1.283	0	1.0258	3.2763	2.25	1.3582	2.1066	2.0205
North East	0.2757	0.3547	1.0258	0	1.4065	0.8622	0.4083	0.5807	0.8647
North West	1.4086	1.123	3.2763	1.4065	0	2.4537	1.5384	1.9762	2.8145
South East	1.2336	1.1485	2.25	0.8622	2.4537	0	1.1736	1.6795	1.7886
South West	0.7676	0.4644	1.3582	0.4083	1.5384	1.1736	0	0.8954	0.7566
West Midlands	0.9277	0.99	2.1066	0.5807	1.9762	1.6795	0.8954	0	1.6715
Yorkshire	1.0356	1.6072	2.0205	0.8647	2.8145	1.7886	0.7566	1.6715	0
	Round 18								
East Midlands	0	0.7966	1.6067	0.2064	0.5919	1.8647	0.6541	0.8852	0.8557
East of England	0.7966	0	2.9413	0.4109	0.9693	2.7044	1.4794	0.4601	0.6676
London	1.6067	2.9413	0	0.7219	1.8714	7.3971	3.4288	2.1196	1.4498
North East	0.2064	0.4109	0.7219	0	0.4535	1.1267	0.3679	0.2336	0.2159
North West	0.5919	0.9693	1.8714	0.4535	0	1.872	0.8594	0.578	0.6607
South East	1.8647	2.7044	7.3971	1.1267	1.872	0	2.7444	1.7606	2.3518
South West	0.6541	1.4794	3.4288	0.3679	0.8594	2.7444	0	0.8636	0.7494
West Midlands	0.8852	0.4601	2.1196	0.2336	0.578	1.7606	0.8636	0	0.7022
Yorkshire	0.8557	0.6676	1.4498	0.2159	0.6607	2.3518	0.7494	0.7022	0
	BA.1								
East Midlands	0	0.5961	2.2629	0.3726	1.3085	1.3199	0.561	0.9276	1.1549
East of England	0.5961	0	1.9081	0.5842	1.0124	0.926	0.4802	0.8685	1.0013
London	2.2629	1.9081	0	1.0435	3.407	2.1588	1.4827	2.032	2.4728
North East	0.3726	0.5842	1.0435	0	1.0228	0.6441	0.3251	0.6117	0.7404
North West	1.3085	1.0124	3.407	1.0228	0	2.2095	1.4275	1.9152	2.1833
South East	1.3199	0.926	2.1588	0.6441	2.2095	0	1.222	1.5149	1.9063
South West	0.561	0.4802	1.4827	0.3251	1.4275	1.222	0	1.2322	0.5397
West Midlands	0.9276	0.8685	2.032	0.6117	1.9152	1.5149	1.2322	0	1.4975
Yorkshire	1.1549	1.0013	2.4728	0.7404	2.1833	1.9063	0.5397	1.4975	0
	BA.1.1								
East Midlands	0	0.6169	2.5062	0.3256	1.0662	1.1894	0.9603	1.6355	0.4974
East of England	0.6169	0	3.2082	0.3561	1.2404	1.0861	0.7378	1.1974	0.7993
London	2.5062	3.2082	0	2.0594	3.8541	6.3044	3.4978	3.8658	2.4259
North East	0.3256	0.3561	2.0594	0	0.9978	1.3282	0.5091	0.4334	0.574
North West	1.0662	1.2404	3.8541	0.9978	0	1.6379	1.0605	1.3395	1.3799
South East	1.1894	1.0861	6.3044	1.3282	1.6379	0	1.6005	1.7963	1.9378
South West	0.9603	0.7378	3.4978	0.5091	1.0605	1.6005	0	0.7702	0.5839
West Midlands	1.6355	1.1974	3.8658	0.4334	1.3395	1.7963	0.7702	0	0.775
Yorkshire	0.4974	0.7993	2.4259	0.574	1.3799	1.9378	0.5839	0.775	0
	BA.2								
East Midlands	0	1.1577	2.7079	0.3323	0.3626	1.183	0.5458	0.7978	0.3666
East of England	1.1577	0	3.6468	0.3188	0.8431	2.1351	1.3199	0.753	0.513
London	2.7079	3.6468	0	0.7829	2.4499	12.1975	4.5813	2.5276	2.2745
North East	0.3323	0.3188	0.7829	0	0.5284	0.3373	0.3222	0.3421	0.3757
North West	0.3626	0.8431	2.4499	0.5284	0	1.2129	0.7308	0.5033	0.399
South East	1.183	2.1351	12.1975	0.3373	1.2129	0	2.2973	1.1183	1.0511
South West	0.5458	1.3199	4.5813	0.3222	0.7308	2.2973	0	0.4843	0.8688
West Midlands	0.7978	0.753	2.5276	0.3421	0.5033	1.1183	0.4843	0	0.853
Yorkshire	0.3666	0.513	2.2745	0.3757	0.399	1.0511	0.8688	0.853	0
