# Supplementary material for: Dynamics of competing SARS-CoV-2 variants during the Omicron epidemic in England
Source: Nat Commun. 2022 Jul 28;13:4375. doi: 10.1038/s41467-022-32096-4 (PMC9330949; doi:10.1038/s41467-022-32096-4)
Supplement: Supplementary file 3 — Description of Additional Supplementary Files [file 41467_2022_32096_MOESM3_ESM.pdf]

Description of Additional Supplementary Files

File Name: Supplementary Data 1
Description: Accession numbers for sequences collected by REACT-1 over rounds 8 to 19 on GISAID and the European Nucleotide Archive.

File Name: Supplementary Data 2
Description: Acknowledgements table for all authors who contributed to the genetic sequences obtained from GISAID that was used in this work.
