# Supplementary material for: Dynamics of competing SARS-CoV-2 variants during the Omicron epidemic in England
Source: Nat Commun. 2022 Jul 28;13:4375. doi: 10.1038/s41467-022-32096-4 (PMC9330949; doi:10.1038/s41467-022-32096-4)
Supplement: Supplementary file 6 — Reporting Summary [file 41467_2022_32096_MOESM6_ESM.pdf]

nature portfolio

Corresponding author(s): Oliver Eales, Marc Chadeau-Hyam, Paul Elliott, Christl Donnelly

Last updated by author(s): Jun 17, 2022

Reporting Summary

Nature Portfolio wishes to improve the reproducibility of the work that we publish. This form provides structure for consistency and transparency in reporting. For further information on Nature Portfolio policies, see our Editorial Policies and the Editorial Policy Checklist.

Statistics

For all statistical analyses, confirm that the following items are present in the figure legend, table legend, main text, or Methods section.

n/a	Confirmed	
☐	☒	The exact sample size (*n*) for each experimental group/condition, given as a discrete number and unit of measurement
☐	☒	A statement on whether measurements were taken from distinct samples or whether the same sample was measured repeatedly
☐	☒	The statistical test(s) used AND whether they are one- or two-sided<br>*Only common tests should be described solely by name; describe more complex techniques in the Methods section.*
☐	☒	A description of all covariates tested
☒	☐	A description of any assumptions or corrections, such as tests of normality and adjustment for multiple comparisons
☐	☒	A full description of the statistical parameters including central tendency (e.g. means) or other basic estimates (e.g. regression coefficient) AND variation (e.g. standard deviation) or associated estimates of uncertainty (e.g. confidence intervals)
☐	☒	For null hypothesis testing, the test statistic (e.g. *F*, *t*, *r*) with confidence intervals, effect sizes, degrees of freedom and *P* value noted<br>*Give P values as exact values whenever suitable.*
☐	☒	For Bayesian analysis, information on the choice of priors and Markov chain Monte Carlo settings
☒	☐	For hierarchical and complex designs, identification of the appropriate level for tests and full reporting of outcomes
☒	☐	Estimates of effect sizes (e.g. Cohen's *d*, Pearson's *r*), indicating how they were calculated

Our web collection on statistics for biologists contains articles on many of the points above.

Software and code

Policy information about availability of computer code

Data collection	All data collection for the REACT1 study is captured with Questback (Spring 2020 installation, version 35).
Data analysis	Statistical analyses were all conducted using the R software version 4.0.5.<br>Software used in fitting the mixed-effects Bayesian P-spline model is provided in the GitHub Repository: https://github.com/mrc-ide/reactidd with vignettes detailing its use available at: https://github.com/mrc-ide/reactidd/vignettes/TemporalOmicronPaper/<br>Constant growth rate models were implemented in the R package 'brms' (version 2.14.4)<br>Viral genome sequencing was done using the ARTIC protocol (version 4) for viral RNA amplification, CoronaHiT for preparation of sequencing libraries, the ARTIC bioinformatics pipeline and assigned lineages using PangoLEARN (version 2022-02-28).<br>Phyleogeographic models were fitted using IQTree (version 2.1.4-beta) and TreeTime (version 0.8.6) implemented in Python (version 3.7)

For manuscripts utilizing custom algorithms or software that are central to the research but not yet described in published literature, software must be made available to editors and reviewers. We strongly encourage code deposition in a community repository (e.g. GitHub). See the Nature Portfolio guidelines for submitting code & software for further information.

Data

Policy information about availability of data

All manuscripts must include a data availability statement. This statement should provide the following information, where applicable:

- Accession codes, unique identifiers, or web links for publicly available datasets
- A description of any restrictions on data availability
- For clinical datasets or third party data, please ensure that the statement adheres to our policy

Access to REACT-1 individual-level data is restricted to protect participants' anonymity.

Summary statistics and data , descriptive tables, and code including the daily weighted number of tests, weighted number of positive tests and daily number of Delta, BA.1, BA.1.1 and BA.2 samples (used for the P-spline models) from the current REACT-1 study are available at https://github.com/mrc-ide/reactidd (DOI:https://doi.org/10.5281/zenodo.6557251).

Sequence read data are available without restriction from the European Nucleotide Archive at https://www.ebi.ac.uk/ena/browser/view/PRJEB37886, and consensus genome sequences are available from the Global initiative on sharing all influenza data (GISAID). The accession numbers are provided in the supplementary data.

Requests for materials should be made to Paul Elliott, p.elliott@imperial.ac.uk, School of Public Health, Imperial College London, Norfolk Place, London, W2 1PG. Aggregate data can only be shared if there is an appropriate number of individuals in each category such that data remains unidentifiable. For more information on the questions that are asked to participants (data variables available) please refer to the REACT-1 study materials (https://www.imperial.ac.uk/medicine/research-and-impact/groups/react-study/for-researchers/react-1-study-materials/). Response to requests should normally be received within a month of the request being made.

Human research participants

Policy information about studies involving human research participants and Sex and Gender in Research.

Reporting on sex and gender	Information on the sex of participants was collected. Sex based analysis was not performed for this study as the population of England has significant mixing between sexes, and so trends in prevalence over time should be the same over the duration of this study.
Population characteristics	See below
Recruitment	We obtained a random population sample of adults in England, using the National Health Service (NHS) patient list, which includes name, address, age and sex of everyone registered with a general practitioner (and therefore is close to the entire UK population). Participants were invited to take part in the study via letters sent in the post. There may have been biases in the individuals who agreed to participate in the study following an initial invitation. Particularly more health conscious individuals may have been more likely to participate, these individuals might have been less likely to be infected with SARS-CoV-2 due to different behavior. We investigated differential response rates by age, sex, ethnicity and social factors. These were similar to those regularly observed in population surveys.
Ethics oversight	We obtained research ethics approval from the South Central-Berkshire B Research Ethics Committee (IRAS ID: 283787).

Note that full information on the approval of the study protocol must also be provided in the manuscript.

Field-specific reporting

Please select the one below that is the best fit for your research. If you are not sure, read the appropriate sections before making your selection.

☐ Life sciences ☒ Behavioural & social sciences ☐ Ecological, evolutionary & environmental sciences

For a reference copy of the document with all sections, see nature.com/documents/nr-reporting-summary-flat.pdf

Behavioural & social sciences study design

All studies must disclose on these points even when the disclosure is negative.

Study description	REACT-1 is a series of community prevalence surveys including virological swabs and reverse transcriptase polymerase chain reaction (RT-PCR) tests from a series of age-sex stratified representative population samples of 100,000 to 150,000 individuals in England. The age range is 5 years and above.
Research sample	In each round of the (n=18) rounds of the study, individuals are sampled from the NHS patient list. In order to achieve the required sample size of 100,000 to 150,000 per rounds , up to 750,000 individuals aged 5 years and above were randomly selected and sent

personalised invitations. For children (5 to 17 years old) the invitation is sent via parents/guardians. In some rounds the sample size obtained missed the target sample size; the lowest sample size obtained for a given round was 94,950. The NHS patient list is close to the entire population of England and so the individuals randomly selected to be invited to participate are a form a representative sample of the population of England. The individuals who agreed to participate in the study and thus form the research sample were less representative of the population of England due to differential response rates between age, sex, ethnicity and social factors, but the differential response rates were similar to those regularly observed in population surveys.

Sampling strategy	Age and sex stratified representative samples of 100,000 to 150,000 were drawn randomly from the general population of England. Samples are stratified by lower-tier local authority to achieve numbers of participants in each area representative of the population size in each area. Conservative sample size calculations estimated that 150,000 and 100,000 participants would ensure the detection, at a 95% confidence level, of PCR positivity prevalence of 1.2 and 1.7%, respectively.
Data collection	Study participants were sent by post test kits and instructions. This included a self-administered throat and nasal swab, and completion of a short online or telephone questionnaire informing on demographic, behavioral factors and recent symptoms. A parent or guardian took the swab for children aged 12 years or below and helped completing the questionnaire. The swab was sent by courier or post to one of the national COVID-19 testing centres or to a commercial laboratory. Researchers were blind to the testing conditions for the self-administered swab tests that were performed.
Timing	The sample collection of the REACT-1 study started in May 2020, the dates of data collection for rounds 14 to 18 were:<br>Round 14: 9 September 2021 - 27 September 2021<br>Round 15: 19 October 2021 - 5 November 2021<br>Round 16: 23 November 2021 - 14 December 2021<br>Round 17: 5 January 2022 - 20 January 2022<br>Round 18: 8 February 2022 - 1 March 2022
Data exclusions	No data were excluded from the analysis
Non-participation	The 3.96 millions registration letters sent in rounds 14 to 18 resulted in 0.49 million (12.5%) registered participants, of whom, 70% successfully completed their test and follow-up questionnaires.
Randomization	Participants were not allocated to experimental groups

Reporting for specific materials, systems and methods

We require information from authors about some types of materials, experimental systems and methods used in many studies. Here, indicate whether each material, system or method listed is relevant to your study. If you are not sure if a list item applies to your research, read the appropriate section before selecting a response.

Materials & experimental systems

n/a	Involved in the study	
☒	☐	Antibodies
☒	☐	Eukaryotic cell lines
☒	☐	Palaeontology and archaeology
☒	☐	Animals and other organisms
☒	☐	Clinical data
☒	☐	Dual use research of concern

Methods

n/a	Involved in the study	
☒	☐	ChIP-seq
☒	☐	Flow cytometry
☒	☐	MRI-based neuroimaging
